# Differential abundance and transcription of 14-3-3 proteins during vegetative growth and sexual reproduction in budding yeast

**DOI:** 10.1038/s41598-018-20284-6

**Published:** 2018-02-01

**Authors:** Ravinder Kumar

**Affiliations:** 1 Department of Biosciences and Bioengineering, Indian Institute of Technology Bombay, Powai, Mumbai-400 076 Maharashtra India; 2 Present Address: Section of Molecular Biology, Division of Biological Science, University of California San Diego, La Jolla, California 92093-0322 USA

## Abstract

14-3-3 is a family of relatively low molecular weight, acidic, dimeric proteins, conserved from yeast to metazoans including humans. Apart from their role in diverse cellular processes, these proteins are also known for their role in several clinical implications. Present proteomic and biochemical comparison showed increased abundance and differential phosphorylation of these proteins in meiotic cells. Double deletion of *bmh1*^−/−^*bmh2*^−/−^ leads to complete absence of sporulation with cells arrested at G_1_/S phase while further incubation of cells in sporulating media leads to cell death. *In silico* analysis showed the presence of 14-3-3 interacting motifs in bonafide members of kinetochore complex (KC) and spindle pole body (SPB), while present cell biological data pointed towards the possible role of yeast Bmh1/2 in regulating the behaviour of KC and SPB. We further showed the involvement of 14-3-3 in segregation of genetic material and expression of human 14-3-3β/α was able to complement the function of endogenous 14-3-3 protein even in the complex cellular process like meiosis. Our present data also established haplosufficient nature of *BMH1/2*. We further showed that proteins synthesized during mitotic growth enter meiotic cells without *de novo* synthesis except for meiotic-specific proteins required for induction and meiotic progression in *Saccharomyces cerevisiae*.

## Introduction

14-3-3 s are a group of acidic, relatively low molecular weight proteins conserved in all eukaryotic species studied so far. These proteins were first identified in the brain^1^ while their significance was first recognised when these proteins were known for their involvement in the synthesis of neurotransmitter^2^. Since then, these proteins have been implicated in diverse cellular process^3^. Apart from their role in normal cellular physiology, these proteins are also found to be associated with several medical implications *viz* Miller–Dieker syndrome and spinocerebellar ataxia type 1, bovine spongiform encephalopathy (BSE)^4,5^, Alzheimer^6^, cancer^7–9^, bacterial^10,11^, viral infection^12–14^ and diabetes^15,16^.

Recent studies over the span of last decade showed the important role of 14-3-3 proteins in the cell cycle. For example, it has been observed that budding yeast Bmh1 also functions as spindle position checkpoint^17^ and yeast 14-3-3 play an important role in DNA damage and spindle checkpoint^18^. Apart from a role in mitotic cell cycle, many studies showed the involvement of these proteins in sexual reproduction or meiosis. For example, 14-3-3η was found to be essential for normal meiotic spindle formation during *in vitro* maturation of mouse oocytes^19^ while 14-3-3ε interacts with the phosphorylated form of Cdc25B and helps in the release of mouse oocyte from prophase I arrest^20^ and all the isoforms of 14-3-3 were reported in mammalian eggs^21^. In *S. pombe*, Rad24p (fission yeast homolog of 14-3-3) acts as a negative factor for meiosis by antagonising the function of meiRNA to promote the formation of a nuclear Mei2p dot^22^ and *A. thaliana* 14-3-3 were able to complement *rad24* in *S. pombe*^23^. Recent proteomics study also showed an increased abundance of yeast 14-3-3 proteins in meiotic cells^24^ while the levels of these proteins were similar in quiescent cells^25^.

Thus taken together with data published from different labs and our own proteomics studies compelled us to further check the level and expression profile of these proteins in mitosis, meiosis and also tested the role of these proteins in the cell cycle especially in meiosis. Our present iTRAQ based quantitative proteomics data showed that both yeast 14-3-3 showed increased abundance in meiosis despite the low rate of transcription, translation, and the active proteasome. Further, our gel-based phosphoproteomics data showed differential phosphorylation of both Bmh1 and Bmh2 in mitosis and meiosis while transcriptomics data showed reduced expression of yeast 14-3-3 during meiosis. Our data also showed the ability of human 14-3-3 beta/alpha in rescuing meiotic defects in *bmh1*^−/−^ *bmh2*^+/−^ and *bmh2*^−/−^*bmh1*^+/−^ strains. We further showed the possible involvement of Bmh1 and Bmh2 in regulating the behaviour of spindle pole body and kinetochore complex. Our present data also shows the important role of 14-3-3 proteins in the maintenance of cell viability during stress condition. Thus our present study shows differential abundance, phosphorylation, and expression of yeast 14-3-3 proteins in mitotic and meiotic cells along with an important role in duplication, segregation of genetic material and direct involvement of 14-3-3 in the induction and meiotic progression.

## Results

### Differential abundance of yeast 14-3-3 proteins in mitosis and meiosis

**Figure 1:**
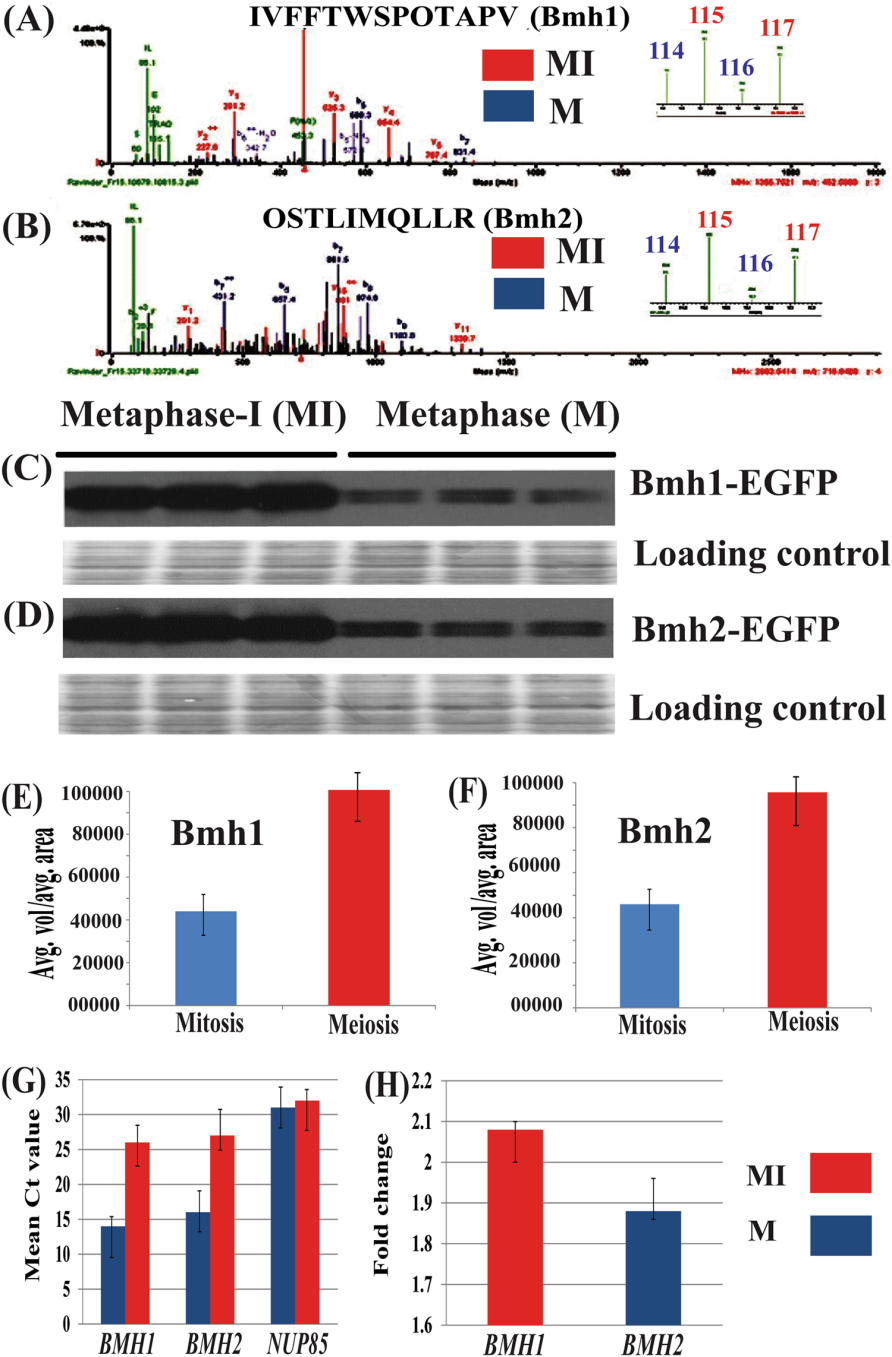
Cellular abundance and transcription pattern of budding yeast 14-3-3 proteins in mitosis and meiosis. Spectra of one of the peptide of (**A**) *Bmh1 and (**B**) Bmh2 along with the intensity of reporter ions. Western blot and densitometry analysis showing increased abundance of Bmh1-EGFP (**C**,**E**) and Bmh2-EGFP (**D**,**F**) in meiosis (left) when compared to mitosis (right). *Western blot and densitometry analysis were also performed in a previous study^24^. (**G**) Bar graph showing mean Ct values of *BMH1, BMH2* in mitosis (metaphase, purple bar) and meiosis (metaphase-I, red bar) along with *NUP85* used as internal control for calculating fold change. (**H**) Fold change of *BMH1* (red bar) and *BMH2* (purple bar). Equal loading of protein in each well was confirmed by Ponceau S staining of a membrane.

In our earlier report of gel-based proteomics (2-DE and 2D-DIGE) of mitosis (mitotic metaphase) and meiosis (metaphase-I)^24^, we showed the differential abundance of proteins in these two types of the cell cycle. Due to the limitation of gel-based proteomics we were able to detect only around 26 unique proteins which showed differential abundance during the vegetative growth phase and sexual reproduction. Thus to overcome the limitations associated with gel based proteomic platforms (including gel to gel variation, pH range, detection of low abundant proteins) and to increase the proteome coverage we performed iTRAQ based quantitative proteomics^26^. By using iTRAQ, a gel-free proteomic platform we were able to detect total 690 proteins (in biological triplicate) with 1% FDR (False discovery rate) and 60% SPI (Scored peak intensity) thus putting high stringency during data analysis using SpectrumMill from Agilent. Identified proteins were grouped based on biological processes, molecular functions, cellular components and distribution of proteins into a different class to understand their biological relevance (Fig. S1). Out of hundreds of proteins detected in iTRAQ based quantitative proteomics, we focussed our attention on budding yeast 14-3-3 proteins as these proteins followed the same trend in present iTRAQ analysis (Fig. 1A,B), previous gel based analysis^24^ and due to their involvement in cell cycle as mentioned in the introduction. We further validate our iTRAQ data using western blot and denstiometric analysis by detecting Bmh1-EGFP (Fig. 1C,E and Fig. S2A,B) and Bmh2-EGFP (Fig. 1D,F and Fig. S2A,B) using anti-GFP antibodies (using same biological triplicate which were used in iTRAQ based proteomic analysis mentioned above). Proteins which are routinely used as loading controls also showed differential abundance in mitosis and meiosis (example actin, tubulin, Glyceraldehyde 3-phosphate dehydrogenase), as a result, we are showing Ponceau S stained blot showing equal loading of proteins and specificity of anti-GFP antibodies were checked separately. Our western blot data support our previous proteomics data as well as our present iTRAQ data. Our previous gel based^24^ and present gel free iTRAQ based quantitative proteomics along with western blot data showed an increased abundance of 14-3-3 proteins in meiosis suggesting that these proteins may have the relatively more important role(s) in sexual reproduction.

Surprisingly, apart from budding yeast 14-3-3, hundreds of proteins showed increased abundance in meiosis despite the fact that there was a reduced level of transcription, translation in meiosis (except for meiotic-specific proteins) and active proteasomal machinery^27^.

### Expression of yeast 14-3-3 proteins

Further to check the correlation between protein abundance and mRNA level of Bmh1 and Bmh2, we first compared the mRNA abundance of *BMH1* and *BMH2* in mitosis and meiosis. Total RNA was extracted from metaphase and metaphase-I arrested cells (biological triplicate) and cDNA were prepared for RT-qPCR as described previously^24^. Our present RT-qPCR data showed that level of *BMH1* and *BMH2* mRNA is less in meiosis compared to mitosis and have almost similar fold change (Fig. 1G, H). *NUP85* (NUclear pore) whose level remains similar in mitosis and meiosis^24,28^ was taken as an internal control and for calculating the fold change (Fig. 1H). Our RT-qPCR data was supported by previous studies^29^ which showed degradation of mRNA as cells progress in meiosis. Our RT-qPCR data contradicts our proteomics data and we observed opposite trend for both Bmh1 and Bmh2 at protein and mRNA level.

Apart from Bmh1 and Bmh2, we previously observed negative correlation for Cof1, Act1 between protein abundance and mRNA in meiosis^24^ suggesting that this trend is not unique to Bmh1 or Bmh2 but maybe with other proteins too thus making it a global phenomenon.

### Increased stability of yeast 14-3-3 in meiosis

In our previous experiment, we demonstrated a negative correlation between our proteomics and transcriptomic data for both *BMH1* and *BMH2*. We also got similar results for *COF1* and *ACT1* (as mentioned above) suggesting that this behaviour is not specific to Bmh1 and Bmh2 alone but maybe with other proteins which showed increased abundance in meiosis. The negative correlation between protein and mRNA raises two possibilities. One possibility is that protein gets accumulated during the last phase of pre-meiotic growth regime and same proteins enter meiosis without any *de novo* translation in meiosis (except for meiotic-specific proteins like Mam1, Rec8) while mRNA degraded over a period during the meiotic regime. Another possibility is that cells accumulated proteins after fresh translation from the same mRNA again and again in meiosis.

**Figure 2:**
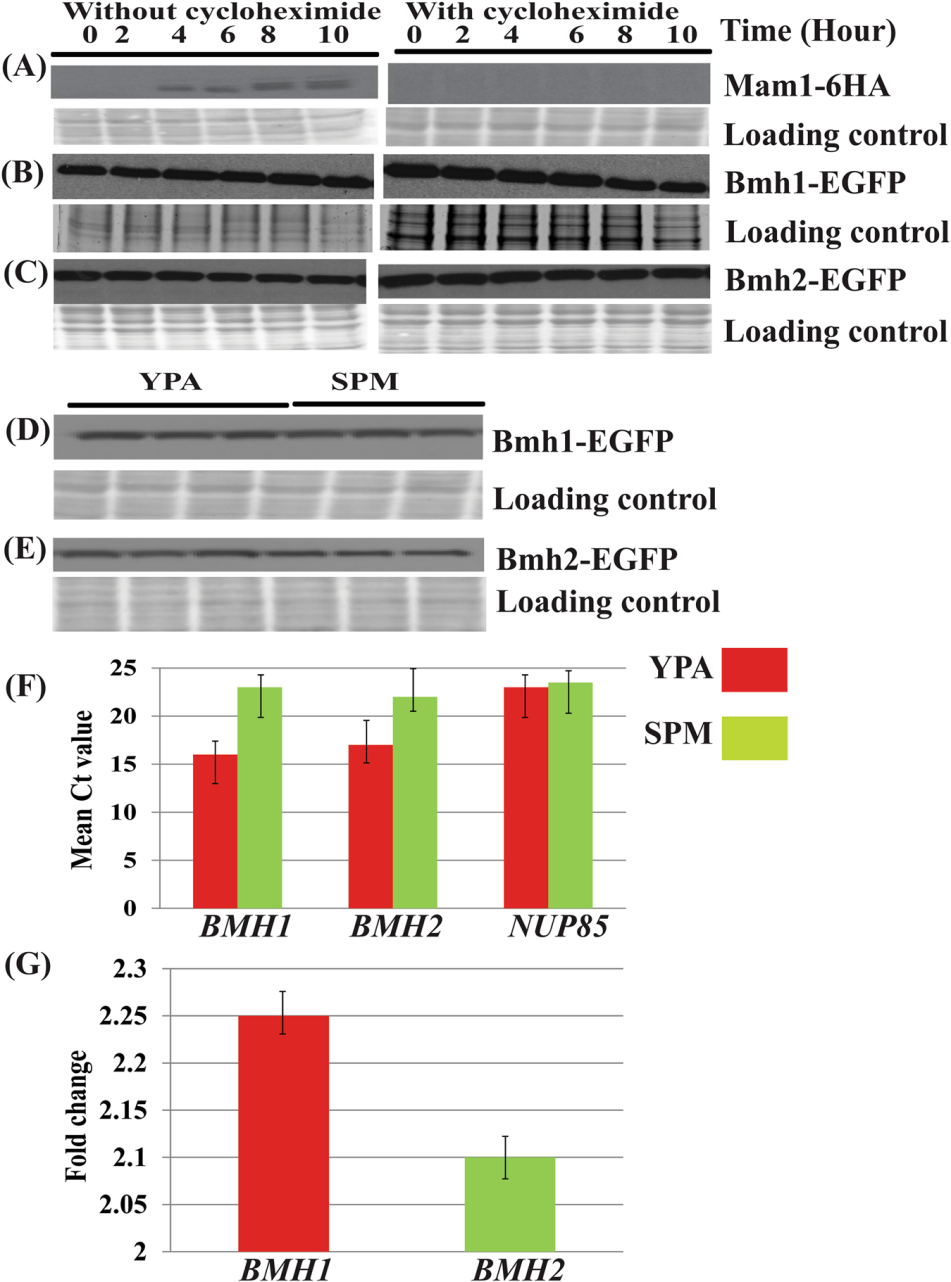
Increased stability of budding yeast *Bmh1 and Bmh2 in meiosis. Effect of cycloheximide on a translation of (**A**) Mam1-6HA (used as a control). (**B**) Bmh1-EGFP (**C**) Bmh2-EGFP in absence (left) and in presence (right) of cycloheximide in sporulation media. Samples were collected at indicated time points and cycloheximide was added just after the release of cells in the sporulating medium. Level of (**D**) Bmh1-EGFP (**E**) Bmh2-EGFP in YPA (left) and SPM (right). Ponceau S stained blot was used as loading control in all the cases. *Protein stability for this protein was also checked in the previous study^24^. (**F**) Bar graph showing mean Ct values of *BMH1, BMH2* of cells from pre-meiotic (red bar) and meiotic culture (green bar) along with *NUP85* used as an internal control for calculating fold change. (**G**) Fold change of *BMH1* (red bar) and *BMH2* (green bar).

We used cycloheximide, a potent eukaryotic translation inhibitor^30^, to check the first possibility where proteins were translated and accumulated during terminal stage of pre-meiotic growth regime in YPA. We treated cells with 20 µg/mL cycloheximide immediately after releasing the cells in sporulating medium and proteins were extracted at indicated time points (Fig. 2). We failed to find any significant difference in levels of Bmh1 and Bmh2 in treated and untreated cultures supporting the assumption that proteins synthesised during the later stage of pre-meiotic regimes enter into meiosis with no fresh translation of these proteins. (Fig. 2B,C and Fig. S2D and Fig. S2E respectively). Cycloheximide activity in meiosis was checked by inhibiting translation of meiosis-specific protein Mam1-6HA^31^. Mam1-6HA was detected using anti-HA antibodies (Fig. 2A and Fig. S2C). Present western blot data suggests that whatever proteins were detected even after 10 hour of cycloheximide treatment was synthesised before adding cycloheximide i.e. before culture was transferred to sporulating media. Further cycloheximide treatment also ruled out the possibility of *de novo* or fresh translation of Bmh1 and Bmh2 in meiosis, again suggesting that whatever proteins (at least for Bmh1 and Bmh2) were there in meiosis were coming from mitotic cells of the pre-meiotic culture. We further compared the level of Bmh1/2 in cells harvested from the pre-meiotic (YPA) and sporulating media (SPM). Our western blot data showed that level of Bmh1 (Fig. 2D) and Bmh2 (Fig. 2E) were similar in the pre-meiotic and meiotic cells. Full blot images for these figures are also shown in Fig. S2F. Thus taken together data of present western blot and protein stability together support our hypothesis that cells enter into the meiotic mode with same proteins which were synthesised in cells during pre-meiotic growth regime without any fresh translation except for meiotic-specific proteins.

We further compared the mRNA levels of *BMH1* and *BMH2* in the pre-meiotic and meiotic cultures. Our RT-qPCR results indicate the level of *BMH1* and *BMH2* mRNA was more in YPA (pre-meiotic) compared to SPM (meiotic) cultures. (Fig. 2F,G). Further, fold change of *BMH1* and *BMH2* mRNA in pre-meiotic culture was close to one another. This shows that mitotic cells both in YPD and YPA have more mRNA compared to meiotic cells in SPM. *NUP85* was used as internal control and for normalisation of data for calculation of fold change^28^.

### Differential phosphorylation of 14-3-3 in meiosis

**Figure 3:**
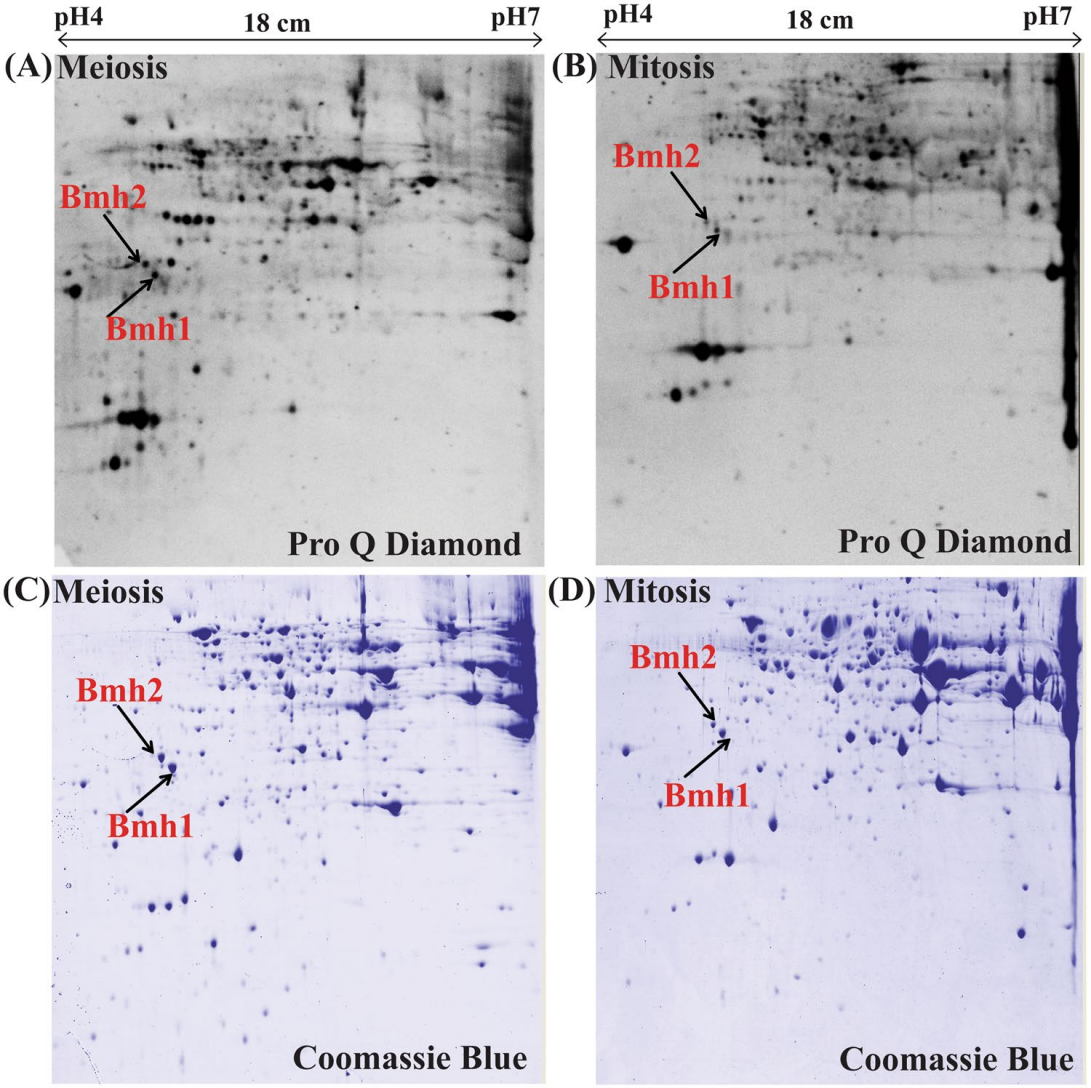
Differential phosphorylation of budding yeast Bmh1 and Bmh2 in meiosis. ProQ Diamond stained 2-DE gels (**A**) meiosis (**B**) mitosis, same gels were later stained with coomassie brilliant blue (CBB) for total proteome (**C**) meiosis and (**D**) mitosis.

In the previous experiment, we showed an increased cellular abundance of Bmh1 and Bmh2 in meiosis while both Bmh1 and Bmh2 along with Act1 and Cof1^24^ showed a negative correlation between mRNA and protein. It is also known that both Bmh1 and Bmh2 are phosphoproteins^32–35^ and interaction between 14-3-3 and their interacting partners are almost always phosphorylation dependent^35,36^. Thus we checked the level of Bmh1 and Bmh2 phosphorylation in meiosis and mitosis using 2-DE gels stained with phospho-specific ProQ Diamond stain^34^. ProQ Diamond staining of 2-DE gels showed differential phosphorylation of 14-3-3 in mitosis and meiosis (Fig. 3). Apart from phosphorylation, yeast 14-3-3 proteins also showed a post-translation modification in the form of ubiquitination, acetylation, and succinylation^32–35,37,38^ although the biological implication of these post-translational modification remains elusive and whether these modifications play any role in altering protein stability needs further research. Therefore  it will be interesting to compare ubiquitination, acetylation and succinylation level of yeast 14-3-3 protein in mitosis and meiosis. Apart from that, it will be important and interesting to map and compare phosphorylation pattern of Bmh1/2 in mitosis and meiosis.

### Growth curve, sporulation efficiency, spore viability and chromosome segregation analysis of yeast 14-3-3 mutants

To dissect the biological significance of increased abundance of Bmh1 and Bmh2 in meiosis, we generate several mutants of yeast 14-3-3 encoding genes. A null mutant of either *bmh1*^−/−^ or *bmh2*^−/−^ alone does not have any significant effect on growth, sporulation efficiency and spore viability. Even meiotic progression was similar in wild-type and *bmh1*^−/−^ or *bmh2*^−/−^ mutant alone (data not shown) and our data is supported by a recent study^39^.

**Figure 4:**
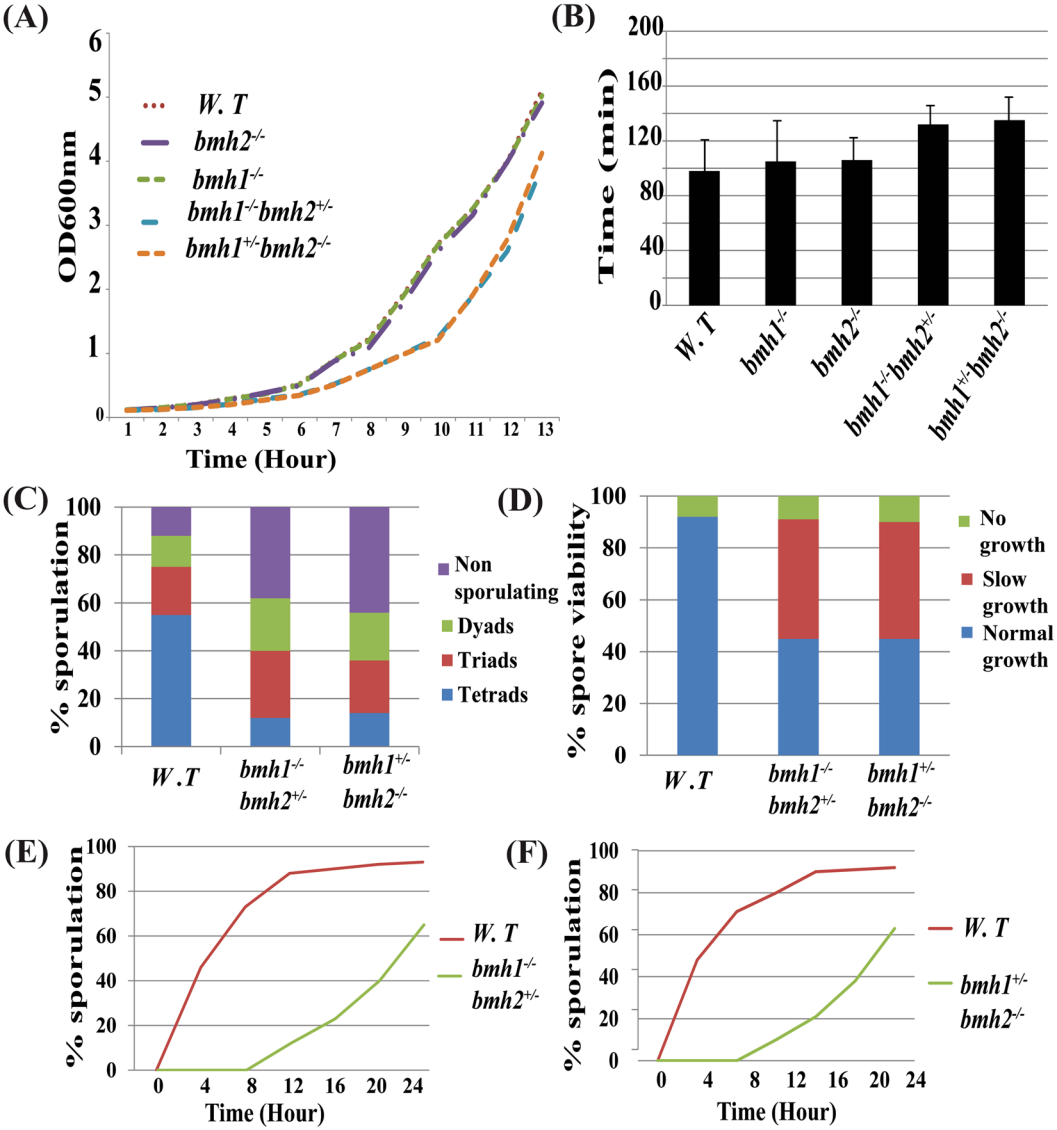
Growth curve, sporulation efficiency, spore viability of *bmh1*^−/−^*bmh2*^+/−^ and *bmh1*^+/−^*bmh2*^−/−^ mutants. (**A**) Growth curve for wild type, *bmh1*^−/−^, *bmh2*^−/−^, *bmh1*^−/−^*bmh2*^+/−^ and *bmh1*^+/−^*bmh2*^−/−^ mutants. Histogram showing (**B**) doubling time (in min), (**C**) sporulation efficiency and (**D**) spore viability in *bmh1*^−/−^*bmh2*^+/−^ and *bmh1*^+/−^*bmh2*^−/−^ mutants along with control. For spore viability, forty tetrads were dissected in each case. Each experiment was performed thrice; data is shown for only one set. Graph showing meiotic progression in (**E**) *bmh1*^−/−^*bmh2*^+/−^ and (**F**) *bmh1*^+/−^*bmh2*^−/−^ mutants along with wild type control. Note each experiment was performed thrice, data is shown for one set. Note: Doubling time was calculated using growth curve in a biological triplicate one set of which is shown in Fig. 4A.

We further went one step ahead and generate *bmh1*^−/−^*bmh2*^+/−^ and *bmh1*^+/−^*bmh2*^−/−^ mutants. In these strains, we observed a dramatic reduction in growth rate, a significant increase in doubling time and decrease in sporulation efficiency (Fig. 4A,B and C respectively). Although, spore viability from these strains showed that all four spores were viable but the spores grow at a different rate (Fig. 4D). Out of four spores, two spores showed normal growth while remaining two spores grows very slowly (Fig. S3B,C). Further, we checked the genome of small spores and found that small spores lack both *BMH1* and *BMH2* (data not shown) together suggesting that slow growth of two spores may be due to the absence of both the 14-3-3 genes. So far it is known that double deletion of *BMH1* and *BMH2* is lethal in most of the background except for Σ1278^40^. Our present work suggests that double deletion is not lethal even in SK1 background and our results are in accordance with a recent study^39^.

Although there was no significant difference in spore viability in wild-type and *bmh1*^−/−^*bmh2*^+/−^ and *bmh1*^+/−^*bmh2*^−/−^ mutants conversely we observed a significant difference in time taken by *bmh1*^−/−^*bmh2*^+/−^ and *bmh1*^+/−^*bmh2*^−/−^ mutants for completion of sporulation compared to wild-type. Compared to wild-type control, in which sporulation cycle completes within 14 hours of incubation in the sporulating medium while mutant strains sporulate later in 24 hours after release in sporulating media (Fig. 4E,F). Apart from that, we observed increased percentage of dyads and triads in *bmh1*^−/−^*bmh2*^+/−^ and *bmh1*^+/−^*bmh2*^−/−^ mutants compared to wild-type or *bmh1*^−/−^ or *bmh2*^−/−^ respectively suggesting the possible role of 14-3-3 proteins in meiotic progression.

### FACS analysis of *bmh1*^−/−^*bmh2*^−/−^

From the earlier experiment, we observed that double deletion of *bmh1*^*−*^*bmh2*^*−*^ is not lethal even in our SK1 background and this allowed construction of yeast strain null for both *BMH1* and *BMH2*. Using this strain, we checked the sporulation efficiency and we observed a severe defect in sporulation in *bmh1*^−/−^*bmh2*^−/−^ strain (Fig. S3D,E) and we fail to detect sporulation in *bmh1*^−/−^*bmh2*^−/−^ strain suggesting the important role of 14-3-3 in sexual reproduction or meiosis. It is important to mention that similar to previous study^39^ we could not get any growth of *bmh1*^−/−^*bmh2*^−/−^ in YPA. Therefore we directly collected biomass of wild-type and *bmh1*^−/−^*bmh2*^−/−^ strain from YPD plate and release cells in sporulating medium and samples were collected for analysis at indicated time point (the detailed procedure is mentioned in materials and methods section).

**Figure 5:**
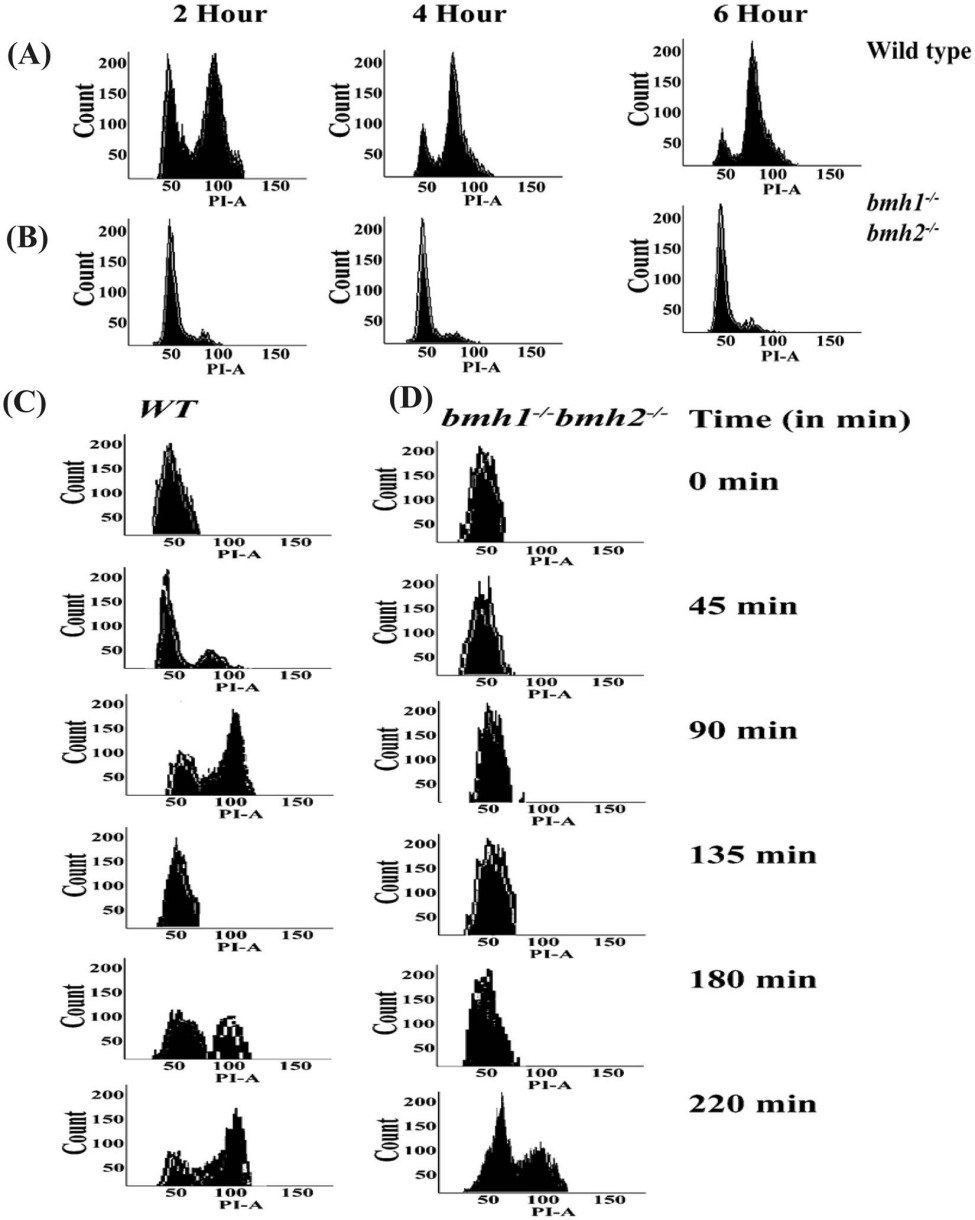
Bmh1 and Bmh2 together play a role in DNA duplication. FACS analysis of control cells (**A**) wild-type cells and (**B**) *bmh1*^−/−^*bmh2*^−/−^ mutants in the sporulating medium. For FACS analysis samples were collected at indicated time points. FACS analysis showing DNA duplication in (**C**) wild-type haploid and (**D**) *bmh1*^*−*^*bmh2*^*−*^ mutant strain.

We further checked the stage(s) at which *bmh1*^−/−^*bmh2*^−/−^ cells arrested in sporulating media. Earlier reports with 14-3-3 suggest that these proteins play an important role in initiation and elongation of DNA replication. Hence we first performed FACS analysis to check whether *bmh1*^−/−^*bmh2*^−/−^ cells are able to duplicate their DNA content. Our FACS analysis for samples collected at 2^nd^, 4^th^ and the 6^th^ hour from SPM suggests that DNA duplication is hampered in *bmh1*^−/−^*bmh2*^−/−^ strain suggesting that cells arrest at G_1_/S transition phase of cell cycle (Fig. 5B). This result is in accordance with a previous study^41^ which shows the involvement of these proteins in G_1_/S transition.

Since it is unlikely that cells remain arrested at the G_1_/S stage for a long time; as a result, we checked the viability of *bmh1*^−/−^*bmh2*^−/−^ cells after 5-7 days using propidium iodide staining. Our propidium iodide staining experiment suggests that cells which stuck at G_1_/S phase remain viable for 2-3 days (checked by trypan blue as well as by propidium iodide, Fig. S3G just after release of cells in SPM) and then starts dying and most of the cells died after 7-8 days (Fig. S3H 5^th^ day from the release of cells into SPM) while the wild-type cells completed sporulation well before completion of one day. PI-stained wild-type cells just after release in SPM is shown in Fig. S3F. This experiment also signifies that budding yeast 14-3-3 proteins also play important role in the viability of cells under nutrient scarcity.

### Role of 14-3-3 in DNA duplication and chromosome segregation

Previous studies from different labs^41,42^ and data from the last experiment showed that 14-3-3 proteins are important in DNA duplication. We took advantage of the fact that double deletion is not lethal even in SK1 background and perform FACS analysis even for mitotic cells. Compared to wild-type control, *bmh1*^*−*^*bmh2*^*−*^ strains took a long time for DNA duplication and cells spends substantial time in S phase (Fig. 5D) compared to wild-type control (Fig. 5C). Our present FACS data is in accordance with a previous study^41,42^ which also showed the role of 14-3-3 proteins in DNA duplication.

**Figure 6:**
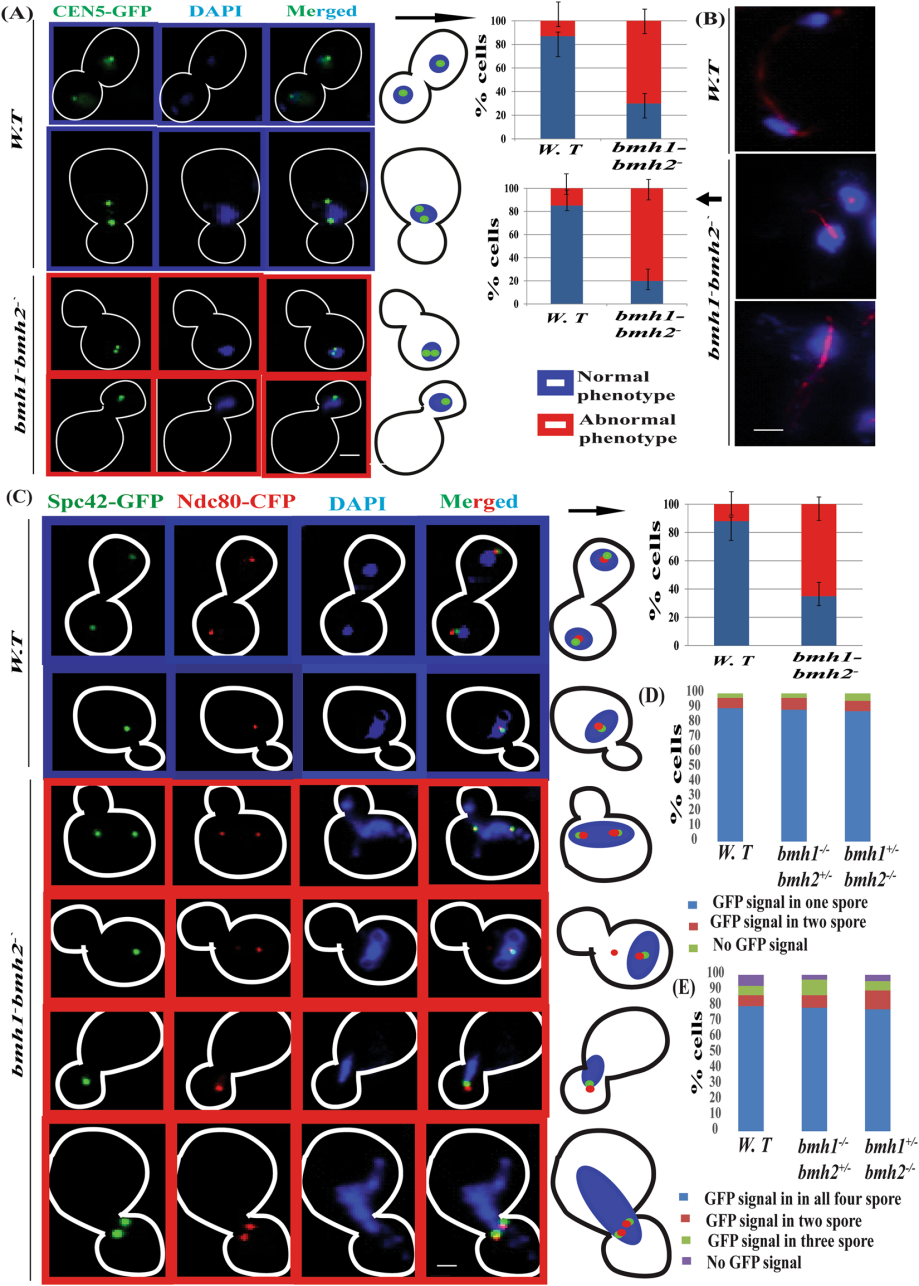
Involvement of budding yeast 14-3-3 proteins in chromosome segregation. (**A**) Chromosome segregation in wild type strain [panel 1 and 2 {blue}] and *bmh1*^*−*^*bmh2*^*−*^ mutant strain [panel 3, 4, {red}]. Scale bar = 2 µM. (**B**) Positioning and length of spindle in wild type (top) and *bmh1*^*−*^*bmh2*^*−*^ (middle and bottom) Scale bar = 5 µM. (**C**) Localization of Spc42-GFP and Ndc80-CFP in (wild type strain [panel 1 and 2 {blue}) and in *bmh1*^*−*^*bmh2*^*−*^ (panel 3, 4, 5 and 6 {red}). Scale bar = 2 µM. (**D**) Segregation of homologous chromosome pairs and, (**E**) segregation of sister chromatids in wild-type and various Bmh1/2 mutants respectively. Note for chromosome segregation analysis only tetrads were counted. And each experiment was performed three time and minimum 120 tetrads were checked while the data is shown for one set. Note due low signal intensity of CFP we use false colour in image (signal of CFP is shown in red colour). In each case, minimum 100 cells were counted for statistical analysis. Arrow pointing towards histogram associated with respective panels.

Further to clarify the role of yeast 14-3-3 proteins in chromosome segregation we generated *bmh1*^*−*^*bmh2*^*−*^ GFP-TetR/TetO system to check chromosome segregation in haploid strain. Compared to wild-type strain with normal chromosome segregation (here normal chromosome segregation means that after duplication one chromosome remains in mother-bud while other goes into daughter bud) (Fig. 6A top two panels) we observed significant abnormality in chromosome segregation in *bmh1*^*−*^*bmh2*^*−*^ strains (Fig. 6A bottom two panels) (here abnormal segregation means that after duplication both chromosomes remains either in mother-bud or both move to daughter bud). Thus our present data suggest that budding yeast 14-3-3 proteins do play important role in both DNA replication as well as in chromosome segregation. It is important to mention that we used only large budded cells in the calculation for checking chromosome segregation defect. Apart from that we tagged Mtw1 (a central component of kinetochore) with GFP (MTW1-GFP) in both wild-type and mutant strain and follow a pattern of chromosome segregation and we gain got results similar to with GFP-TetR/TetO or CEN5-GFP (Fig. S4) again confirming the role of 14-3-3 in segregation of genetic material.

After observing gross abnormality associated with chromosome segregation, we check the spindle position in these cells. Our microscopic data showed significant abnormality in terms of length of spindles (Fig. 6B middle and bottom panel) in *bmh1*^*−*^*bmh2*^*−*^ compared to wild-type control (Fig. 6B top panel). This observation further suggests the involvement of 14-3-3 in regulating chromosome segregation and thus making 14-3-3 proteins an important element in proper segregation of genetic material.

### Yeast 14-3-3 as a regulator of kinetochore complex and spindle pole body

It is known that yeast 14-3-3 or Bmh1/2 interact with their interacting partners through conserved signature sequence or motifs [RX(2-3)*pS/pT*XP, where X can be any amino acid residue and *pS/pT* represents phosphorylated serine or threonine]^43^. Thus we checked the presence of Bmh1/2 interacting motifs in bonafide members of spindle pole body and kinetochore complex. Our *in silico* analysis showed the presence of 14-3-3 interacting motifs in members of kinetochore complex and spindle pole body (Table 1, Fig. S5A). The presence of these motifs suggests that 14-3-3 may be involved in modulating the behaviour of these proteins complex which is important in segregation of genetic material during the cell cycle. To check this possibility we tagged C-terminal of Ndc80, component of outer kinetochore complex with CFP and Spc42, the component of spindle pole body with GFP in haploid *bmh1*^*−*^*bmh2*^*−*^ and wild-type strain. Our fluorescence microscopic data showed that there was gross chromosomal segregation defects in mutant strain compared to wild-type, thereby suggesting that budding yeast 14-3-3 proteins may play important role in regulating either localisation, a timely splitting of kinetochore complex as well as spindle pole body (Fig. 6C). Compared to wild-type cell where the fluorescent signal was present in both daughter and mother-bud (Fig. 6C) we observed abnormality in fluorescence signal in mutant strain compared to wild-type (term normal and abnormal phenotype or segregation are already defined in the previous section). We observed four type of phenotype which was predominant in the cell population (Fig. 6C). One where fluorescence (Ndc80-CFP and Spc42-GFP) was present in daughter bud (Fig. 6C), in the mother bud only (Fig. 6C), in mother-bud but opposite to neck (Fig. 6C), two signals in mother or daughter bud only (Fig. 6C). Combined result from four types of abnormality which predominate accounts around 70% of total large budded cells. Previous experimental data also showed the interaction between Ndc80 and 14-3-3^44^. To get more confidence on our data we tagged C- terminal of Iml3 protein with GFP (as a control, as it was previously shown to interact with budding yeast 14-3-3 proteins and also possess 14-3-3 interacting motif, mentioned above)^43,44^. Just like Ndc80-CFP, we observed gross abnormality in the localisation of kinetochore complex in *bmh1*^*−*^*bmh2*^*−*^ using Iml3-GFP (data not shown). Our present data is supported by a recent study which also showed the involvement of 14-3-3 proteins in the positioning of spindle pole body and segregation of genetic material^17^. It is important to mention that chromosome segregation defect was similar to one observed with CEN5-GFP in the previous experiment (Fig. 6A) again highlighting the likely involvement of 14-3-3 proteins in faithful segregation of genetic endowment of a cell.

**Table 1:** List of bonafide members of kinetochore complex and spindle pole body proteins having 14-3-3 interacting motifs.

S. No	Protein symbol^*^	Motif	Signature sequence^**^	Complex name
01	Tub4	RDDDTKP	RXXXTXP	Spindle Pole Body
02	Spc98	RSMVSSP, RQLSNP	RXXXSXP, RXXSXP	Spindle Pole Body
03	Nud1	RNNPITTP	RXXXXTXP	Spindle Pole Body
04	Spc29	RRTSSP	RXXSXP	Spindle Pole Body
05	Mps3	RKVLLSKP	RXXXXSXP	Spindle Pole Body
06	Cdc31	RSSLQSGP	RXXXXSXP	Spindle Pole Body
07	Sfi1	RSDSSP	RXXSXP	Spindle Pole Body
08	Ctf19	RDHTYP	RXXTXP	Kinetochore
09	Dsn1	RMETIP	RXXTXP	Kinetochore
10	Mps1	RSSSRSHP	RXXXXSXP	Kinetochore
11	Bub1	RLIYRTAP	RXXXXTXP	Kinetochore
12	Ctf13	RIQTPP, RLQSLP, RDESSSP	RXXTXP RXXXSXP	Kinetochore
13	Sli15	RLSSIP, RKSSIP	RXXSXP	Kinetochore
14	Dam1	RLSIGSAP	RXXXXSXP	Kinetochore
15	Iml3	RVPSDP	RXXSXP	Kinetochore

^*^Protein symbol as given in SGD (http://www.yeastgenome.org).

^**^In RX(2-3)pT/pSXP, X can be any amino acid residue, pT/pS represents phosphorylated threonine and serine respectively.

Unlike other eukaryotes, budding yeast microtubule organisation center (MTOC) or spindle pole bodies are not free structures which remain embedded firmly in nuclear membrane throughout the cell cycle and therefore undermining the exact role of 14-3-3 in regulating the kinetochore complex and MTOC. And therefore it will be interesting to study the localisation of microtubule organisation center in absence of 14-3-3 proteins in other model systems like fission yeast. Information generated from such a system will certainly be of much help in understanding the role of 14-3-3 in the biology of not only microtubule organisation center but spindles also.

### Role of 14-3-3 in chromosome segregation in meiosis

Our previous results showed the involvement of budding yeast 14-3-3 in chromosome segregation in mitosis. Based on the results of previous experiments we investigate the role of Bmh1/2 in segregation of sister chromatids and homologous chromosomes in meiosis. For this TetO/TetR-GFP on chromosome V of wild-type as well as *bmh1*^+/−^*bmh2*^−/−^ and *bmh1*^−/−^*bmh2*^+/−^ was developed^45–47^. Our cell biological data showed that segregation of sister chromatids, as well as homologous chromosomes, were similar in wild-type as well as in mutant strains (Fig. 6D,E). Our present data showed that either yeast 14-3-3 does not play a role in chromosomes segregation in meiotic cells or even the presence of the single copy of either *BMH1* or *BMH2* in the absence of other isoforms are sufficient to cater the need of cells even in meiosis. Thus data from present and previous sections showed that even a single copy of either isoform of budding yeast 14-3-3 in the complete absence of other isoform can cater the need of cell both during mitosis as well as during meiosis. This further pointed towards overlapping as well as redundant nature of budding yeast Bmh1/2 in mitosis as well as in meiosis. Chromosome segregation analysis could not be studied in *bmh1*^−/−^*bmh2*^−/−^ strains as we failed to get any sporulation in these cells (Fig. S3E).

### Expression of human 14-3-3 β/α in *bmh1*^−/−^*bmh2*^+/−^ and *bmh1*^+/−^*bmh2*^−/−^

**Figure 7:**
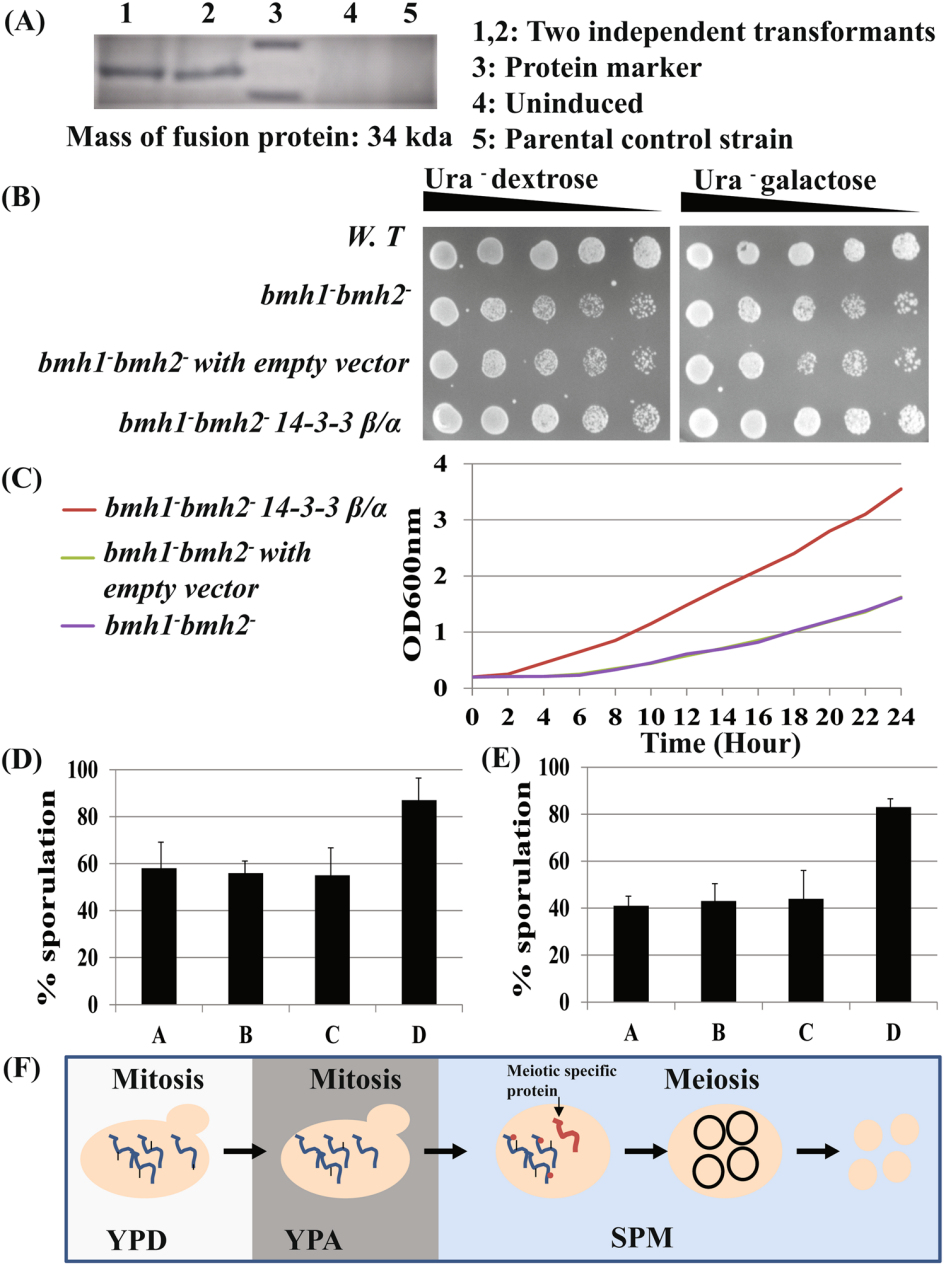
Expression of human 14-3-3 β/α complementing budding yeast *BMH1/2* in mitotic and meiotic cells. (**A**) Expression of human 14-3-3 beta/alpha-6HA in required strain. (**B**) Frogging assay showing the effect of expression of human 14-3-3 β/α along with vector control and *bmh1*^*−*^*bmh2*^*−*^ strain alone. (**C**) Growth curve of *bmh1*^*−*^*bmh2*^*−*^ alone, with 14-3-3 beta/alpha and vector control. Experiment was done three time, data is shown for one set. Expression of human 14-3-3 β/α in (**D**) *bmh1*^−/−^*bmh2*^+/−^ (**E**) *bmh1*^+/−^*bmh2*^−/−^ mutants. {A-empty vector in dextrose, B -14-3-3 in dextrose, C -empty vector in galactose and D-14-3-3 in galactose}. Sporulation percentage was calculated after 24 hour of incubation in the sporulating medium. (**F**) Yeast cells entered into a meiotic programme with proteins synthesized during mitotic growth in YPA.

It is known that 14-3-3 proteins are highly conserved in eukaryotes both at genetic as well as protein level. For instance, human 14-3-3ƞ is 70% identical to budding yeast Bmh1 at amino acid composition^48^. Apart from this, our *in silico* data showed that motifs involved in the interaction of 14-3-3 proteins with their interacting partners are also conserved, thus suggesting that the expression of 14-3-3 from one species into another species can rescue the defect caused due to the deletion of an endogenous 14-3-3 gene^3^. Fig. S5B showing the amino acid sequence alignment of human 14-3-3 beta/alpha along with *S. cerevisiae* Bmh1/2. Expression of human 14-3-3 beta/alpha in required yeast strains was checked by detecting fusion protein of approx. 34 kDa using anti-HA antibodies (Fig. 7A and Fig. S2G for complete blot image) (as 14-3-3 beta/alpha-6HA). We first checked whether expression of human 14-3-3 β/α can rescue the growth defect in *bmh1*^*−*^*bmh2*^*−*^. Our present frogging assay and growth curve data showed that expression of human 14-3-3 β/α can rescue the growth defects in mitotic cells (Fig. 7B,C respectively) and our this observation is in accordance with the previous study^49^. Although we rescued mitotic growth defect, whether expression of human 14-3-3 β/α can also rescue meiotic defect remains uncertain. Successful expression and rescue off mitotic defect compelled us to express human 14-3-3 β/α (under gal promoter) in *bmh1*^−/−^ *bmh2*^+/−^*, bmh2*^−/−^*bmh1*^+/−^ strains. Our present data suggest that expression of human 14-3-3 β/α was able to increase the sporulation in *bmh1*^−/−^ *bmh2*^+/−^*, bmh2*^−/−^*bmh1*^+/−^ compared to empty vector control suggesting that human 14-3-3 can complement yeast 14-3-3 not only in mitosis but also in a specialised complex process like meiosis (Fig. 7D,E). We believe that this is probably the first study where budding yeast meiotic defect was rescued by expression of the heterologous 14-3-3 protein. We observed around 90% sporulation in gal induced culture (from both *bmh1*^−/−^*bmh2*^+/−^ *and bmh1*^+/−^*bmh2*^−/−^) compared to empty vector or uninduced culture where sporulation was around 60% and 50% in *bmh1*^−/−^*bmh2*^+/−^ and *bmh1*^+/−^*bmh2*^−/−^ respectively. Apart from that we also found the decreased percentage of dyads and triads on the expression of human 14-3-3β/α in *bmh1*^−/−^*bmh2*^+/−^ and *bmh1*^+/−^*bmh2*^−/−^ compared to uninduced or vector control (data not shown). Thus our present data showed that expression of human 14-3-3 β/α was able to rescue defects even in meiotic cells. Further, it will be interesting to check whether expressions of other 14-3-3 isoforms can rescue mitotic as well as meiotic defects.

## Discussion

By using gel-free iTRAQ based quantitative proteomics; we were able to identify proteins which showed differential abundance in cells arrested at mitotic metaphase (mitosis) and metaphase -I of meiosis (meiosis). Proteins identified in this gel-free proteomic approach can be classified together in various groups which can help in analysing and understanding the biological process in which these proteins are implicated. It is surprising to see that out of close to seven hundred proteins identified in this gel free quantitative proteomics analysis, a couple of hundred proteins showed increased abundance in meiosis. Why these proteins showed increased abundance in meiosis despite the fact that the proteasome remains active, low translation and degradation of mRNA in meiosis remains an open question. How proteins remain stable in meiosis remains elusive and needs further investigation. Whether some kind of post-translation modification is responsible for observed abundance and stability in meiosis again remains an open question. Our present results of protein stability (using cycloheximide) and ProQ Diamond stained 2-DE gels showed the possibility that increased abundance and stability observed in meiosis may involve some kind of PTM (Post-translational modification).

Based on our present and previous proteomics investigation^24^ we proposed a model (Fig. 7F) according to which budding yeast, during transition from a mitotic to meiotic mode, mitotic proteins translated during pre-meiotic growth regime (mitotic growth in YPA) enters into meiotic cells (in SPM) without *de novo* synthesis leaving aside proteins required for induction and meiotic progression until completion of cell cycle. While entering into the meiotic mode, cells somehow stabilized proteins coming from the mitotic cell cycle. Post-translation modification (including phosphorylation, ubiquitination, glycosylation etc) may be important in stabilizing proteins in meiotic cells while the proteasome remains active. Further, it is important to mention that not all mitotic proteins enter into the meiotic mode and cells somehow eliminate the mitotic specific proteins.

The present study neatly showed the important requirement of 14-3-3 proteins in chromosome segregation and in sexual reproduction. Further, the presence of all the 14-3-3 isoforms in germs cells^21^ clearly pointed toward the possible involvement of these proteins in sexual reproduction even in higher eukaryotes. Whether these proteins are involved in any kind of pregnancy or birth-related defects need to be confirmed. Due to the presence of multiple isoforms, technical difficulty of manipulating all isoforms together or in same cell or organism and ethical issues hampered the study of these proteins in higher eukaryotes like mammals. Still, there are many studies which clearly showed the important and critical role of 14-3-3 proteins in multicellular organisms. For example, it is known that interaction between 14-3-3 protein and Raf-1 is important for meiotic maturation of *Xenopus* oocytes^50^. The complex of Rap1/B-Raf/14-3-3 theta protein is important for morphogenetic differentiation of post-meiotic male germ cells^51^. Rad24p (*S. pombe* 14-3-3), regulates the localization of phosphorylated Ste11p^52^ which is required for expression of genes involved in conjugation and meiosis. In fission yeast Rad24 physically interact with Mei2 which prevents Mei2 assembly into nuclear dot thereby antagonizing meiRNA (which is required for pre-meiotic DNA duplication and meiosis-I)^53,54^. These studies showed the critical role of 14-3-3 in decision making between mitosis/meiosis. Further involvement of 14-3-3 in maintaining symmetry and position of SPB^55^ provide significant support to our observation that 14-3-3 are somehow involved in regulating the behaviour of SPBs. Whether the observed defect in chromosome segregation is due to defect in proper localization, duplication, and splitting of SPBs or kinetochore in the absence of 14-3-3 needs further research. Whether 14-3-3 s are required for induction of meiosis and have any role in the initial steps of meiosis (like induction of Ime1) remains an important question.

It is important to mention that 14-3-3 or Bmh proteins interact with hundreds of cellular proteins, therefore it requires further research to be certain that whatever phenotypes discussed in the present study are a direct or indirect consequence of 14-3-3 deletion. This way, present data fueled further research to get a better understanding as far as the role of 14-3-3 proteins are concerned in various phenotypes mentioned in the present study. Present data also shows that the motifs involved in the interaction between 14-3-3 and their interacting partners are conserved from single-celled yeast to multicellular mammals. Although, higher eukaryotes example, vertebrates acquired motifs with consensus sequence absent or not reported in budding yeast proteins. Further budding yeast lacks non-phosphorylated motifs (like ELVLRSESEEKVV) and CRD or cysteine-rich domain (example RSESEEE). Owing to increase in the number of proteins and complex nature of higher eukaryotes it is not strange that cells over eons of evolution acquired more motifs with a different signature sequence. Expression of human 14-3-3 β/α in *Saccharomyces cerevisiae* and rescue of sporulation defect, suggests that these proteins may also be involved in sexual reproduction even in humans. In conclusion, apart from their role in normal physiology, Bmh1, Bmh2 proteins may also play a crucial role in sexual reproduction. Owing to their high degree of similarity both at genetic as well as at the amino acid level, Bmh1 and Bmh2 may have overlapping and highly redundant functions even in meiosis. Our present data from mitosis as well as from meiosis also pointed towards haplosufficient nature of yeast 14-3-3 isoforms.

## Materials and Methods

### Strain, media, and culture conditions

All the strains constructed for this study (Table S1) are isogenic to the SK1 background. For genetic manipulations (gene deletion, promoter shuffling, and C-terminal tagging of proteins), the PCR-based approach was used as described elsewhere^24,56–58^ using appropriate plasmids obtained from Euroscarf. All the genetic manipulations were confirmed by diagnostic PCR analysis. C-terminal protein fusions were verified both by diagnostic PCR and western blotting. Chromosome V was tagged with GFP using GFP-TetR/TetO system as described earlier^45–47^. List of primers used in strain construction are listed in Table S2. Mitotic and meiotic synchronization was performed as explained previously^24^. Growth curve, immunofluorescence was performed as explained earlier^24^.

### Gal induction of human 14-3-3 β/α

Gal induction of human 14-3-3β/α was performed as described elsewhere^49^.

### Protein extraction, IEF, 2-DE gels running

Protein extraction, IEF, 2-DE gel running was performed as described elsewhere^24,59^. In the present study, we rehydrated 18 cm, 4–7 pH IPG strip (from GE) with 600 µg protein in 350 µL of rehydration for 16 hour at room temperature.

### Staining with ProQ Diamond

2-DE gels were stained with ProQ Diamond stain as per manufacturer instructions (Invitrogen #P33300) and ProQ Diamond stained 2-DE gels were scanned as described elsewhere^60^.

### Total proteome staining

Followed by scanning, gels were again rinsed in MiliQ water and incubated with coomassie in staining solution for overnight in gentle shaking condition. Next morning gels were destained as described previously. Spot detection, gel matching, quantitation, and normalization were carried out by Image Master (Version 6.0, Amersham Biosciences). The intensity of each protein spot was normalized to the total intensity of the entire gel image, respectively^24,59^.

### In-gel digestion, MS analysis

After analysis of gels, significant protein spots were marked on 2-DE gel image, and protein spots were excised from the preparatory gel. In-gel digestions, mass spectrometric analysis for protein identification were performed as described previously^24,59^.

### Microscopy

All the images were captured using Zeiss Axio Vision microscope using appropriate filters.

### Western blot, protein stability

Western blot, protein stability was performed as explained previously^24^.

### cDNA synthesis and RT-qPCR

RNA extraction, cDNA synthesis, and RT-qPCR were performed as described previously^24^.

### FACS analysis

Cells of required strains were harvested and washed twice with distilled water. Cells were then resuspended in 1 mL of absolute ethanol and incubated at 4 °C for 14–16 hour. Tubes were then centrifuged at 12000 rpm for 1 min and the supernatant was discarded. Cells were then washed twice in 50 mM citrate buffer pH 8.0 and finally resuspended in 1 mL of citrate buffer pH 8.0. RNAse at the final concentration of 10 µg/mL was added to each tube and incubated at 50 °C for 3 hour. Tubes were then cooled to room temperature and proteinase K (from Promega) at the final concentration of 30 µg/mL was added and tubes were again incubated at 50 °C for 3 hour. Tubes were then centrifuged at 12000 rpm for 1 min and the cell pellet was resuspended in 1 mL of PBS pH 7.5 and stored at 4 °C. Propidium iodide (PI) at the final concentration of 20 µg/mL was added to tubes just 30 min before FACS analysis (using BD FACS Canto^TM^II).

### iTRAQ (Isobaric tags for relative and absolute quantitation)

Protein extraction, quantification, buffer exchange, in solution digestion, protein labelling with iTRAQ reagent(s), off gel fractionation, ziptip was performed as described elsewhere^25,61^. In this study, we use iTRAQ four-plex kits from Sciex. Peptides from metaphase-arrested cells were labelled with 114, 116 and peptides from metaphase-I arrested cells with 115 and 117 labels. *Saccharomyces cerevisiae* was set as taxonomy during analysis of data. Data analysis was performed using Spectrum Mill (from Agilent) as described previously^62^.

### DAPI (4’,6-diamidino-2-phenylindole) staining

A required number of cells were taken in the fresh tube and washed twice with PBS pH 7.2. Cells were resuspended in 70% ethanol for 1 min and centrifuged at 5000 rpm for 1-2 min. Cells were again washed twice with PBS pH 7.2. Finally, cells were resuspended in 50 µL of PBS pH 7.2. DAPI was added to the cell suspension at the final concentration of 5 µg/mL, and content in the tube was mixed well and stored at 4 °C till further microscopic observation.

### Localization of kinetochore and spindle pole body

C-terminal of Spc42, Ndc80 was tagged with GFP and CFP respectively both in wild-type and *bmh1*^*−*^*bmh2*^*−*^ strains. Iml3-GFP was used as a control which was known to interact with budding yeast 14-3-3 protein. Localisation of the signal from fluorescent protein was checked in wild-type and mutant strains after DAPI staining.

### Checking sporulation in *bmh1*^−/−^*bmh2*^−/−^

A patch of wild-type as well as *bmh1*^−/−^*bmh2*^−/−^ strain was made on YPD plate incubated at 30 °C till sufficient biomass appears on the plate. Cells from patches were taken and washed twice with sterile distilled water. Finally, cells were released in 50 mL SPM in 250 mL flask such that initial OD_600 nm_ was close to one and flasks were incubated at 30 °C, 200 rpm. Samples were collected for FACS analysis and checking of cell viability at indicated time points.

## Electronic supplementary material

Supplementary data
