# Supplementary material for: Differential abundance and transcription of 14-3-3 proteins during vegetative growth and sexual reproduction in budding yeast
Source: Sci Rep. 2018 Feb 1;8:2145. doi: 10.1038/s41598-018-20284-6 (PMC5794856; doi:10.1038/s41598-018-20284-6)
Supplement: Supplementary file 1 — Supplementary data [file 41598_2018_20284_MOESM1_ESM.pdf]

Supplementary information

Differential abundance and transcription of 14-3-3 proteins during vegetative growth and sexual reproduction in budding yeast

Ravinder Kumar[*§],

Department of Biosciences and Bioengineering, Indian Institute of Technology Bombay, Powai, Mumbai- 400076, India

[§]**Present address:** Section of Molecular Biology, Division of Biological Science, University of California San Diego, La Jolla, California-92093-0322, USA

[*]**Correspondence:**

Dr. Ravinder Kumar, Department of Biosciences and Bioengineering, Indian Institute of Technology Bombay, Powai, Mumbai- 400076, India, Email: raj86tau@gmail.com

Legends

Figure S1. Bioinformatics analysis of proteins identified in iTRAQ based quantitative comparison of metaphase (mitosis) and metaphase-I (meiosis). (A) Molecular functions (B) Biological processes (C) Protein classes and (D) Cell components.

Figure S2. Complete blot image: For Bmh1-EGFP and Bmh2-EGFP at low (A) and high (B) exposure, (C) for Mam1-6HA in absence and presence of cycloheximide. (D) For Bmh1-GEFP and (E) Bmh2-EGFP in presence and absence of cycloheximide respectively. (F) For level of Bmh1 and Bmh2 in YPA and SPM medium and (G) for expression of human 14-3-3 beta/alpha.

Figure S3. Spore viability of *bmh1$^{-/-}$bmh2$^{+/-}$* and *bmh1$^{+/-}$bmh2$^{-/-}$* mutants. Spore viability for (A) wild-type (B) *bmh1$^{-/-}$bmh2$^{+/-}$* (C) *bmh1$^{+/-}$bmh2$^{-/-}$*. Minimum forty tetrads were dissected for spore viability; images of tetrads dissected on a single plate in each case are shown. Out of four spores, two grew normally while two grew slowly.

Sporulation and viability in *bmh1$^{-/-}$bmh2$^{-/-}$* cells. Sporulation in (D) wild-type and (E) *bmh1$^{-/-}$ bmh2$^{-/-}$*. PI stained (F) wild-type cells and (G) *bmh1$^{-/-}$bmh2* just after release in the sporulating medium. (H) Five days old cells from SPM bright field (left) and propidium iodide (PI) staining with RFP filter (right).

Figure S4. Role of 14-3-3 in chromosome segregation. (A) Segregation of chromosome as checked by tracking kinetochore protein (Mtw1-GFP) in wild type (first panel) and in mutant strain (second to fourth panel). (B) Statistic associated with this microscopic observation. Minimum 100 cells were counted in each case. Bar scale represents 2 μM.

Figure S5. Alignment of human 14-3-3 beta/alpha with Bmh1, Bmh2 and amino acid sequence of some of the yeast proteins having 14-3-3 interacting motifs. (A) Amino acid sequence of few proteins where 14-3-3 interacting motifs are shown in red. (B) Multiple sequence alignments of *S. cerevisiae* and *H. sapiens* Bmh1/2 and 14-3-3 beta/alpha respectively using multalin online software (http://multalin.toulouse.inra.fr/multalin/). Amino acid sequences of proteins were taken from PubMed

Table S1. List of yeast strains used in this study

Table S2. List of primers used for strains construction in this study

Table S1. List of yeast strains used in this study

Name	Description	Genotype	Parent strain
SGY40	AMya/α	*Ura*$^-$, *Leu*$^-$, *His*$^-$, *Trp*$^-$ *Kan*$^-$	
SGY41	AMya	*Ura*$^-$, *Leu*$^-$, *His*$^-$, *Trp*$^-$ *Kan*$^-$	
SGY42	AMyα	*Ura*$^-$, *Leu*$^-$, *His*$^-$, *Trp*$^-$ *Kan*$^-$	
SGY68	*pCLB2-CDC20* (MAT a)	*Ura*$^-$, *Leu*$^-$, *His*$^-$, *Trp*$^-$, *Kan*$^+$	SGY41
SGY69	*pCLB2-CDC20* (MAT α)	*Ura*$^-$, *Leu*$^-$, *His*$^-$, *Trp*$^-$, *Kan*$^+$	SGY42
SGY70	*pCLB2-CDC20* (a/α)	*Ura*$^-$, *Leu*$^-$, *His*$^-$, *Trp*$^-$, *Kan*$^+$	SGY68 X SGY69
SGY236	*OsTIR1* in SG41 (MAT a)	*Ura*$^+$, *Leu*$^-$, *His*$^-$, *Trp*$^-$, *Kan*$^+$	SGY41
SGY237	*OsTIR1* in SG42 (MAT α)	*Ura*$^+$, *Leu*$^-$, *His*$^-$, *Trp*$^-$, *Kan*$^+$	SGY42
SGY242	*CDC20-6HA-AID* (MAT α)	*Ura*$^+$, *Leu*$^-$, *His*$^-$, *Trp*$^-$, *Kan*$^+$	SGY237
SGY262	*CDC20-6HA-AID* (MAT a)	*Ura*$^+$, *Leu*$^-$, *His*$^-$, *Trp*$^-$, *Kan*$^+$	SGY236
SGY261	*CDC20-6HA-AID* (a/α)	*Ura*$^+$, *Leu*$^-$, *His*$^-$, *Trp*$^-$, *Kan*$^+$	SGY236XSGY237
SGY460	*pCLB2-CDC20, BMH1-EGFP* (MAT α)	*Ura*$^-$, *Leu*$^-$, *His*$^-$, *Trp*$^+$, *Kan*$^+$	SGY69
SGY459	*pCLB2-CDC20, BMH1-EGFP* (MAT a)	*Ura*$^-$, *Leu*$^-$, *His*$^-$, *Trp*$^+$, *Kan*$^+$	SGY68
SGY462	*CDC20-6HA-AID, BMH1-EGFP* (MAT α)	*Ura*$^+$, *Leu*$^-$, *His*$^-$, *Trp*$^+$, *Kan*$^+$	SGY242
SGY461	*CDC20-6HA-AID, BMH1-EGFP* (MAT a)	*Ura*$^+$, *Leu*$^-$, *His*$^-$, *Trp*$^+$, *Kan*$^+$	SGY262
SGY465	*CDC20-6HA-AID, BMH1-EGFP* (MAT a/α)	*Ura*$^+$, *Leu*$^-$, *His*$^-$, *Trp*$^+$, *Kan*$^+$	SGY462XSGY461
SGY464	*pCLB2-CDC20 , BMH1-EGFP* (MAT a/α)	*Ura*$^-$, *Leu*$^-$, *His*$^-$, *Trp*$^+$, *Kan*$^+$	SGY460XSG4Y59
SGY800	MAT a *bmh1*$^-$	*Ura*$^-$, *Leu*$^-$, *His*$^-$, *Trp*$^-$ *Kan*$^+$	SGY41
SGY801	MAT α *bmh1*$^-$	*Ura*$^-$, *Leu*$^-$, *His*$^-$, *Trp*$^-$ *Kan*$^+$	SGY42
SGY802	MAT a*/a bmh1*$^{+/-}$	*Ura*$^-$, *Leu*$^-$, *His*$^-$, *Trp*$^-$ *Kan*$^+$	SGY800XSGY801
SGY803	MAT a*/a bmh*$^{-/-}$	*Ura*$^-$, *Leu*$^-$, *His*$^-$, *Trp*$^-$ *Kan*$^+$	SG8Y00XSGY41
SGY804	MAT a *bmh2*$^-$	*Ura*$^-$, *Leu*$^-$, *His*$^-$, *Trp*$^-$ *Kan*$^+$	SGY41
SGY805	MAT α *bmh2*$^-$	*Ura*$^-$, *Leu*$^-$, *His*$^-$, *Trp*$^-$ *Kan*$^+$	SGY42
SGY806	MAT a*/a bmh2*$^{+/-}$	*Ura*$^-$, *Leu*$^-$, *His*$^-$, *Trp*$^-$ *Kan*$^+$	SG8Y04XSGY805
SGY807	MAT a*/a bmh2*$^{-/-}$	*Ura*$^-$, *Leu*$^-$, *His*$^-$, *Trp*$^-$ *Kan*$^+$	SGY804XSGY41
SGY827	MAT a*/a bmh1*$^{-/-}$ *bmh2*$^{+/-}$	*Ura*$^-$, *Leu*$^-$, *His*$^+$, *Trp*$^-$ *Kan*$^+$	SGY803
SGY828	MAT a*/a bmh1*$^{+/-}$ *bmh2*$^{-/-}$	*Ura*$^-$, *Leu*$^-$, *His*$^+$, *Trp*$^-$ *Kan*$^+$	SGY807
SGY829	MAT a*/a bmh1*$^{-/-}$ *bmh2*$^{+/-,}$ Human 14-3-3	*Ura*$^+$, *Leu*$^-$, *His*$^+$, *Trp*$^-$ *Kan*$^+$	SGY827
SGY830	MAT a*/a bmh1*$^{+/-}$ *bmh2*$^{-/-}$ Human 14-3-3	*Ura*$^+$, *Leu*$^-$, *His*$^+$, *Trp*$^-$ *Kan*$^+$	SGY828
SGY833	*bmh1*$^-$*bmh2*$^-$	*Ura*$^-$, *Leu*$^-$, *His*$^+$, *Trp*$^-$ *Kan*$^+$	Spores of SGY827
SGY111	MAT aTetO TetR	*Ura*$^+$, *Leu*$^+$, *His*$^+$, *Trp*$^-$ *Kan*$^+$	SGY836
SGY832	MAT a*/a bmh1*$^{+/-}$ *bmh2*$^{-/-}$ *pESC-URA3*	*Ura*$^+$, *Leu*$^-$, *His*$^+$, *Trp*$^-$ *Kan*$^+$	SGY828
SGY834	*bmh1*$^+$*bmh2*$^-$ SPC42-GFP, NDC80-CFP	*Ura*$^+$, *Leu*$^-$, *His*$^+$, *Trp*$^+$ *Kan*$^-$	SGY1337

SGY840	*bmh1*$^-$*bmh2*$^-$ MTW1-GFP	*Ura*$^+$, *Leu*$^-$, *His*$^-$, *Trp*$^-$ *Kan*$^+$	SGY826
SGY845	*bmh1*$^-$*bmh2*$^-$ *TetO/TetR*	*Ura*$^+$, *Leu*$^+$, *His*$^+$, *Trp*$^-$ *Kan*$^+$	Spores of SGY839
SGY835	*bmh1*$^-$*bmh2*$^-$ SPC42-GFP, NDC80-CFP	*Ura*$^+$, *Leu*$^+$, *His*$^+$, *Trp*$^+$ *Kan*$^+$	SGY834
SGY826	*Bmh1*$^{-/-}$*bmh2*$^{-/-}$	*Ura*$^+$, *Leu*$^-$, *His*$^+$, *Trp*$^-$*Kan*$^+$	SGY827

Table S2. List of primers used for strains construction in this study

Name	Description	Nucleotide sequence
**GM45F**	*CDC20-6HA-AID* tagging	GTGAGATTCATACAAGGAGGCCCTCTAGTACCA GCCAATATTTGATCAGG CGTACGCTGCAGGTCGAC
**GM46R**	*CDC20-6HA-AID* tagging	AAATTTCATTATATGCCTTGACATGAACTTTTAT TTTTTTTATTTTATCA ATCGATGAATTCGAGCTCG
**GM47F**	Diagnostic PCR for *CDC20-6HA-AID* tagging	GGCAAGGAAGGTTGTCG
**GM48R**	Diagnostic PCR for *CDC20-6HA-AID* tagging	GTGTGGTGTGTGGGTTC
**MA23**	*pCLB2-CDC20* promoter shuffle	TTTGATTTTTGTGTCCAATTGGAAAGAAACCCAA AAATATAGAAATCGTCGAATTCGAGCTCGTT TAAAC
**MA24**	*pCLB2-CDC20* promoter shuffle	CGGTTACCGCTAATTGCTGCATTTCCCTTATCTC TAGAGCTTTCTGGCATGCACTGAGCAGCGTA ATCTG
**MA25**	Diagnostic PCR for *pCLB2-CDC20*	GTTAGCTTTCCTTCACTTCC
**MA26**	Diagnostic PCR for *pCLB2-CDC20*	CTCTAGATGTTGTCGGTTGC
**MA27**	Diagnostic PCR for *pCLB2-CDC20* (within *Clb2* promoter)	GCGAGTGCATTAGCACAGTG
**RK5**	Internal control in RT-PCR (*NUP85*)	GATTGGGAACAACCATGC
**RK6**	Internal control in RT-PCR (*NUP85*)	AACGGGCCATAGTTCCTT
**RK15**	C-terminal tagging of *BMH1*	AACATCAGCAACAGCAGCCACCTGCTGCCGCCG AAGGTGAAGCACCAAAGCGTACGCTGCAGG TCGAC
**RK16**	C-terminal tagging of *BMH1*	TTTTTTTTCTTTTTTTTTAGTAATTTCTCTTTAGATT TATCAGAATACTTAATCGATGAATTCGAGCT CG
**RK17**	Diagnostic PCR for *BMH1* tagging	GGTCAAGCTGAAGACCAA
**RK18**	Diagnostic PCR for *BMH1* tagging	CTACAAATTATTACACCCCCG
**RK23**	BMH1 deletion	CGCAAGCAAGTGAGAAGAAAAAGCAAGTTAAA GATAAACTAAAGATAAAAGCCAGCTGAAGC TTCGTACG
**RK24**	BMH1 deletion	CTTTTTTTTTTCTTTTTTTTTAGTAATTTCTCTTTAG ATTTATCAGAATACGGCCACTAGTGGATCTG
**RK25**	Diagnostic for *BMH1* deletion	CGAGACGAACCGTAACATA
**RK26**	Diagnostic for *BMH1* deletion	ACACATATAGACATGTACACGC
**RK29**	Diagnostic for *BMH2* deletion	GCCTCTCCCGGTTTTTAATC
**RK30**	Diagnostic for *BMH1* deletion	TCCCCTTGTATTTCTCAGCG
**RK45**	RT-PCR of B*MH1*	GAGGAGTCCAAGGAGAAG
**RK46**	RT-PCR of *BMH1*	AGCCAAATAACGGTGGTA
**RK47**	RT-PCR of *BMH2*	CTCCTCTTTGGAGGCTTA
**RK48**	RT-PCR of *BMH2*	CAGAGGTCCATAAGGTCA

**GM33**	Diagnostic for MTW1 tagging	GTCCTACGAAAATTGGGGC
**GM34**	Diagnostic for MTW1 tagging	GCTGGCTACAGGATTCG
**GM124**	Diagnostic forSPC42 tagging	GCTGAAGCGTGTCGAAGAAG
**GM125**	Diagnostic for SPC42 tagging	CATTGGAACCGCAGATTGCTAG
**MA91**	Diagnostic for NDC80 tagging	GTAGCGCAAAGAATTGAGATTG
**MA92**	Diagnostic for NDC80 tagging	CTTGCTGGCGGTGAAGATAT

Figure S1

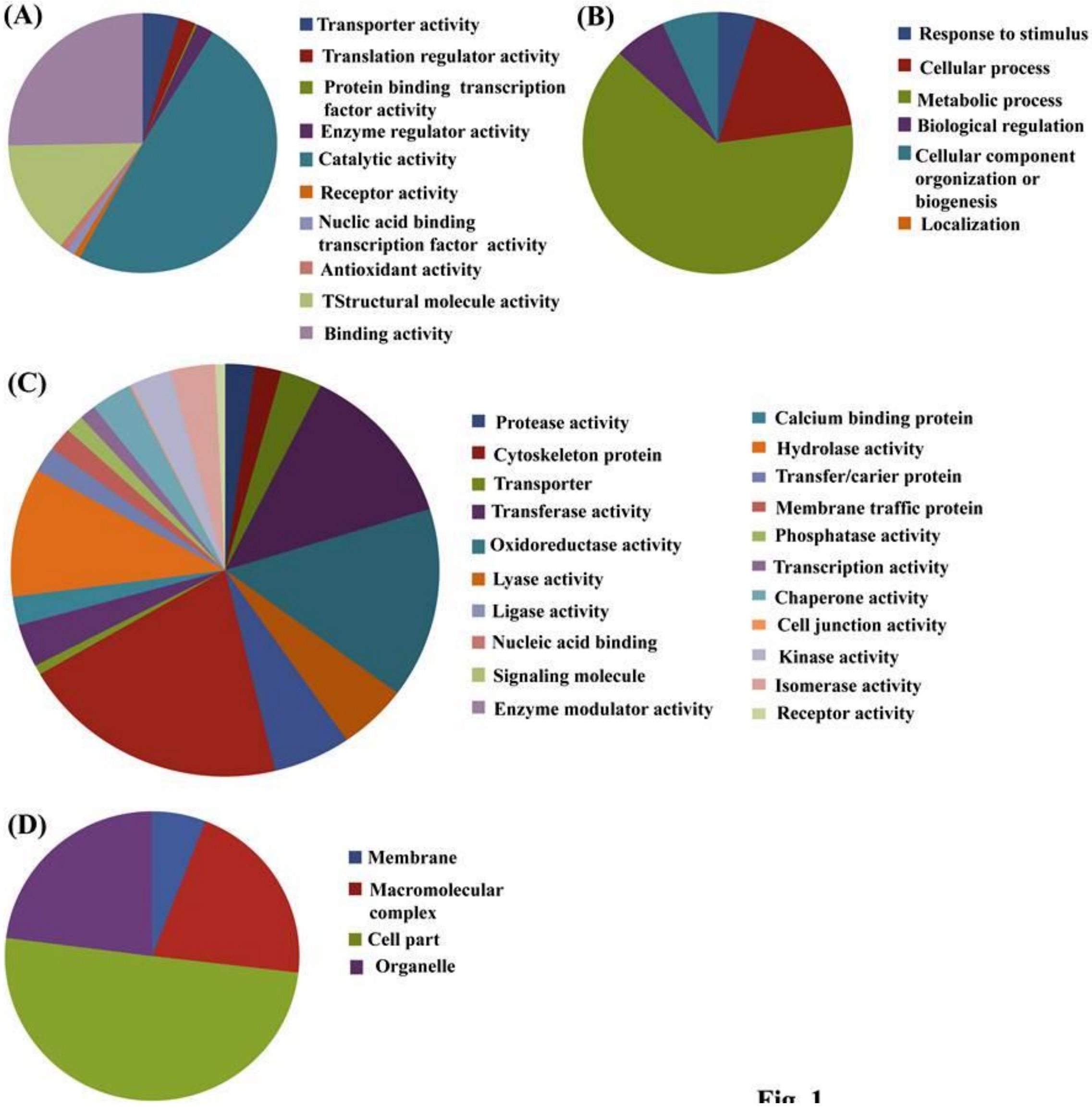
(A)
Transporter activity
Translation regulator activity
Protein binding transcription factor activity
Enzyme regulator activity
Catalytic activity
Receptor activity
Nuclic acid binding transcription factor activity
Antioxidant activity
TStructural molecule activity
Binding activity
(B)
Response to stimulus
Cellular process
Metabolic process
Biological regulation
Cellular component orgonization or biogenesis
Localization
(C)
Protease activity
Cytoskeleton protein
Transporter
Transferase activity
Oxidoreductase activity
Lyase activity
Ligase activity
Nucleic acid binding
Signaling molecule
Enzyme modulator activity
Calcium binding protein
Hydrolase activity
Transfer/carier protein
Membrane traffic protein
Phosphatase activity
Transcription activity
Chaperone activity
Cell junction activity
Kinase activity
Isomerase activity
Receptor activity
(D)
Membrane
Macromolecular complex
Cell part
Organelle
Fig. 1

Figure S2

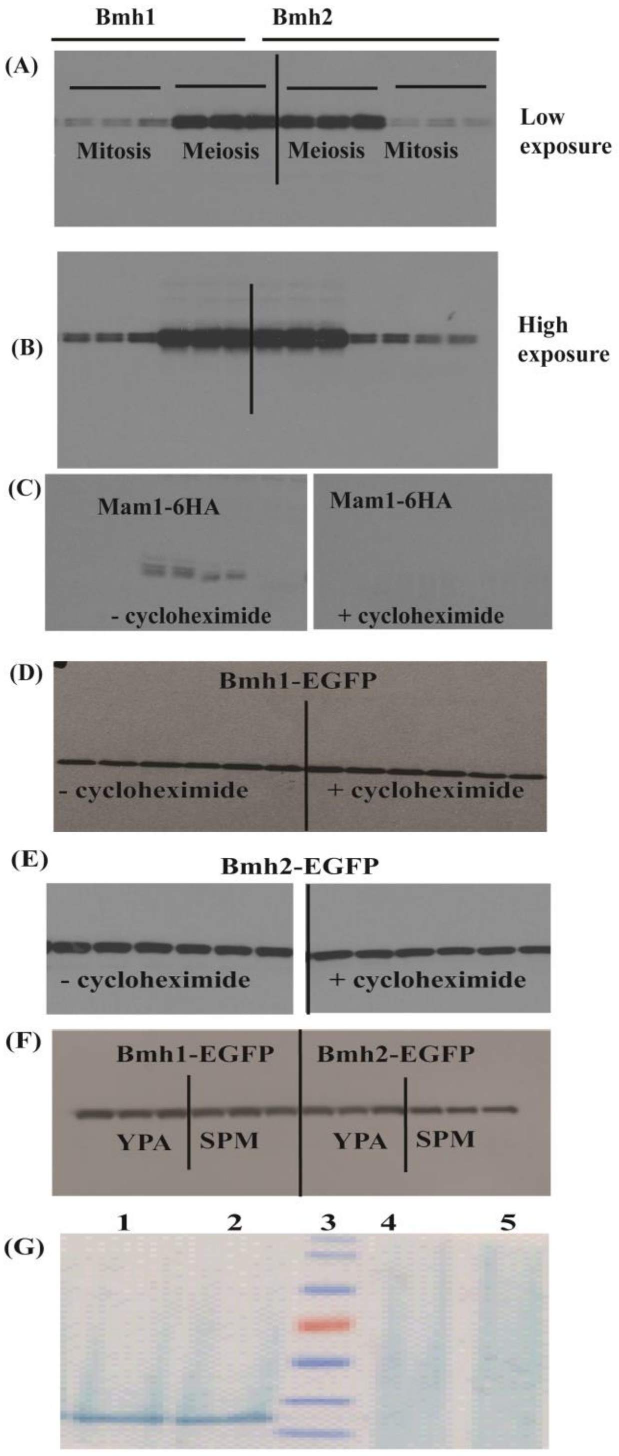

1,2: Two independent transformants
3: Protein marker
4: uninduced
5: parental control strain

Figure S3

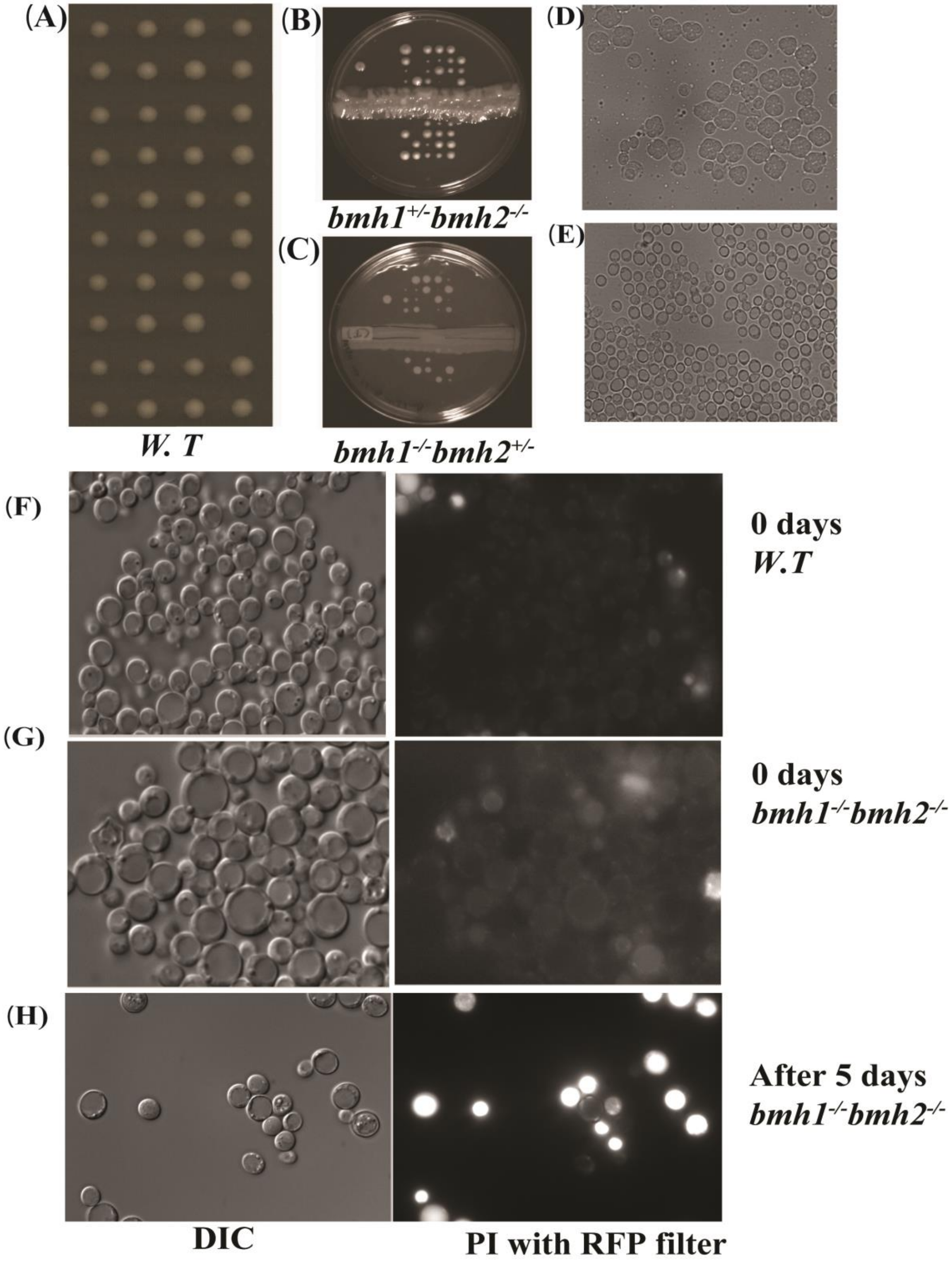
(A)
(B)
(D)
bmh1+/-bmh2-/-
(C)
(E)
W. T
bmh1-/-bmh2+/-
(F)
0 days
W.T
(G)
0 days
bmh1-/-bmh2-/-
(H)
After 5 days
bmh1-/-bmh2-/-
DIC
PI with RFP filter

Figure S4

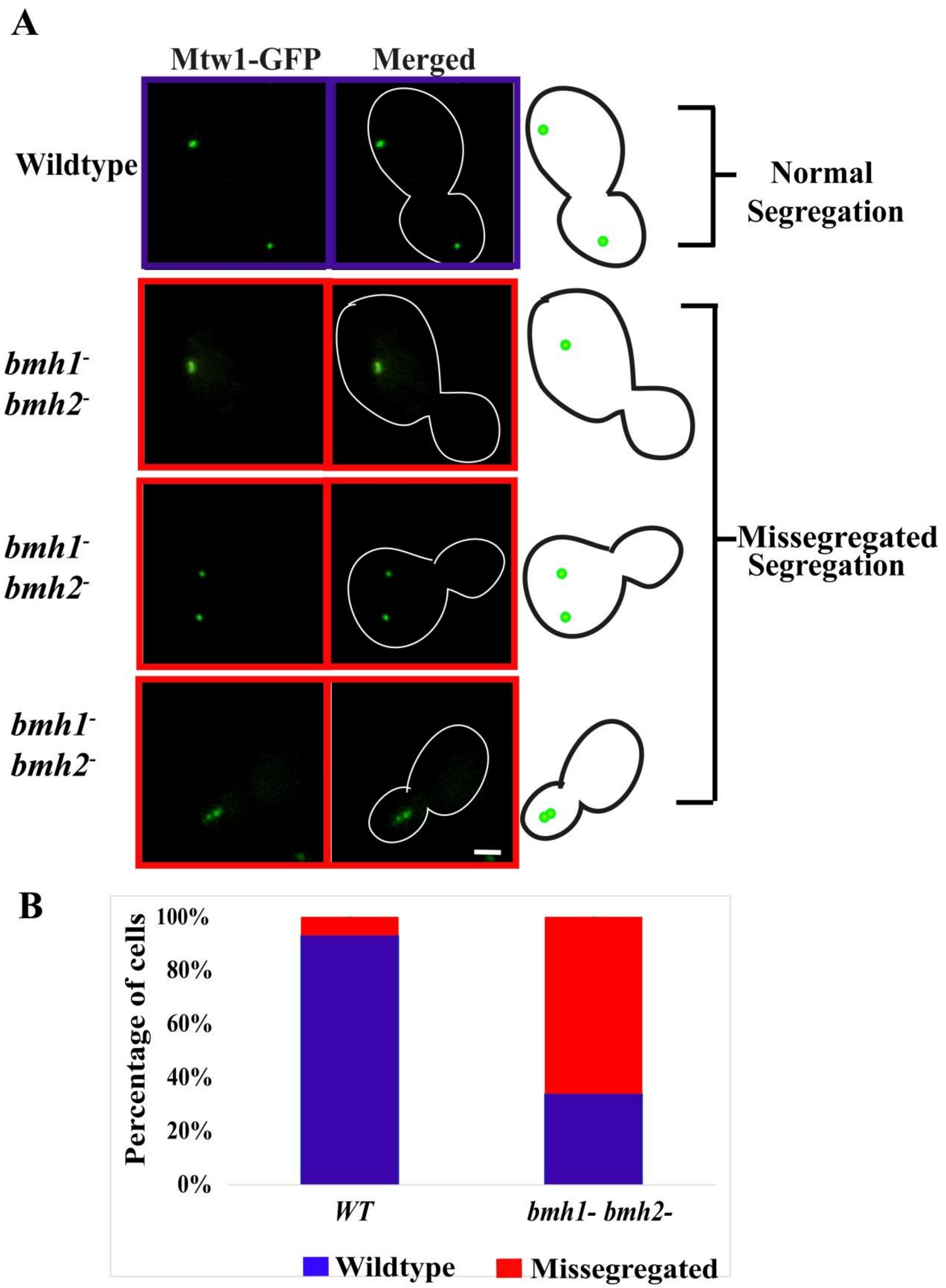
A
Mtw1-GFP
Merged
Wildtype
Normal Segregation
bmh1- bmh2-
bmh1- bmh2-
bmh1- bmh2-
Missegregated Segregation
B
Percentage of cells
100%
80%
60%
40%
20%
0%
WT
bmh1- bmh2-
Wildtype
Missegregated

Figure S5

(A)

Iml3 MPYTWKFLGISKQLSLENGIAKLNQLLNLEVDLDIQTIRVPSDPDGGTAADEYIRYEMRLDISNLDEGTY
SKFIFLGNSKMEVPMFLCYCGTDNRNEVVLQWLKAEYGVIMWPIKFEQKTMIKLADASIVHVTKENIEQI
TWFSSKLYFEPETQDKNLRQFSIEIPRESCEGLALGYGNTMHPYNDAIVPYIYNETGMAVERLPLTSVIL
AGHTKIMRESIVTSTRSLRNRVLAVVLQSIQFTSE

Ctf19 MDFTSDTTNSHDTSNSHLSLENAVGTHHAGEADVNIDGDEKQQLSLLDDDQVRALKLQEEKDALLTRRNT
LLQEIQTYQNILMKENNSKTKNGDILQNDITQDFLNLISISSSNPNSAISDRKRVERINGLTNLQKELVT
KYDTLPLLNMNLRLSYLRDHTYPHLQVSVQSRDRVHNDGIEVLVVNYKFCRNTMNPFEIQFKMFYKFEDS
TLLKWEILRISTNVRLKAKQLLATRNFQKCLLSLYEFDKIKSKKTGIFQNLINLLKRKTRCYLMNNGDSL
IVERVIREGGLTTIKLQINFIITMPGERGKPRNCFLPMSKISIALWKGGERFNQIDLDEICYGLIKEYGV
KTGLKEICNVCLFPDMYAR

Spc29 MDYNNFGNSASKKFQDDTLNRVRKEHEEALKKLREENFSSNTSELGNKKHYRAQERMSSPLHRLSPAGKS
DDRKVKSPLDDKLRRQLREGNTRLPPPPFSSYGMPPTNRSNLDRIRRRTSSPVRTDKFASQNVIDDQRLE
IKYLERIVYDQGTVIDNLTSRITRLESFILNSISDRGDKNFASLEHSRSFSGFPTNKTYGLQMGGLYEND
MPYRRSSDNINKEGAREDRSSQIHIENESTEDILKILSSSFHN

Spc98 MELEPTLFGIIEALAPQLLSQSHLQTFVSDVVNLLRSSTKSATQLGPLIDFYKLQSLDSPETTIMWHKIE
KFLDALFGIQNTDDMVKYLSVFQSLLPSNYRAKIVQKSSGLNMENLANHEHLLSPVRAPSIYTEASFENM
DRFSERRSMVSSPNRYVPSSTYSSVTLRQLSNPYYVNTIPEEDILKYVSYTLLATTSALFPFDHEQIQIP
SKIPNFESGLLHLIFEAGLLYQSLGYKVEKFRMLNISPMKKALIIEISEELQNYTAFVNNLVSSGTVVSL
KSLYREIYENIIRLRIYCRFTEHLEELSGDTFLIELNIFKSHGDLTIRKIATNLFNSMISLYYEYLMNWL
TKGLLRATYGEFFIAENTDTNGTDDDFIYHIPIEFNQERVPAFIPKELAYKIFMIGKSYIFLEKYCKEVQ
WTNEFSKKYHVLYQSNSYRGISTNFFEIINDQYSEIVNHTNQILNQKFHYRDVVFALKNILLMGKSDFMD
ALIEKANDILATPSDSLPNYKLTRVLQEAVQLSSLRHLMNSPRNSSVINGLDARVLDLGHGSVGWDVFTL
DYILYPPLSLVLNVNRPFGRKEYLRIFNFLWRFKKNNYFYQKEMLKSNDIIRSFKKIRGYNPLIRDIINK
LSRISILRTQFQQFNSKMESYYLNCIIEENFKEMTRKLQRTENKSQNQFDLIRLNNGTIELNGILTPKAE
VLTKSSSSKPQKHAIEKTLNIDELESVHNTFLTNILSHKLFATNTSEISVGDYSGQPYPTSLVLLLNSVY
EFVKVYCNLNDIGYEIFIKMNLNDHEASNGLLGKFNTNLKEIVSQYKNFKDRLYIFRADLKNDGDEELFL
LSKSLR

(B)

S. cerevisiae Bmh1
S. cerevisiae Bmh2
H. sapiens 14-3-3 beta/alpha

```
1        10        20        30        40        50        60        70        80        90        100       110       120       130
|--------+---------+---------+---------+---------+---------+---------+---------+---------+---------+---------+---------+---------|
MSTSREDSVYLAKLAEQAERYEEMVENMKTVASSGQELSVEERNLLSVAYKNVIGARRASWRIVSSIEQKEESKEKSEHQVELICSYRSKIETELTKISDDILSVLDSHLIPSATTGESKVFYYKMKGDY
MTMDKSELVQKAKLAEQAERYDDMAAAMKAVTEQGHELSNEERNLLSVAYKNVVGARRSSWRVISSIEQKTERNEKKQQMGK---EYREKIEAELQDICNDVLELLDKYLIPNATQPESKVFYLKMKGDY
Msmdre#lVqlAKLAEQAERY##MaaaMKaVaeqGqELSnEERNLLSVAYKNV!GARRaSWR!!SSIEQKeErnEKk#qqge...eYReKIEaELqdIc#D!LelLDkhLIPnATqgESKVFYlKMKGDY

131      140       150       160       170       180       190       200       210       220       230       240       250       260
|--------+---------+---------+---------+---------+---------+---------+---------+---------+---------+---------+---------+---------|
HRYLAEFSSGDAREKATNASLEAYKTASEIATTELPPTHPIRLGLALNFSVFYYEIQNSPDKACHLRKQAFDDAIAELDTLSEESYKDSTLIMQLLRDNLTLWTSD-MSESGQAEDQQQQQQHQQQQPPA
FRYLSEVASGDNKQTTVSNSQQAYQEAFEISKKEMQPTHPIRLGLALNFSVFYYEILNSPEKACSLAKTAFDEAIAELDTLNEESYKDSTLIMQLLRDNLTLWTSENQGDEGDAGEGEN
hRYLaEfaSGDar#katnaSq#AYqeAfEIakkE$qPTHPIRLGLALNFSVFYYEIqNSP#KAChLaKqAFD#AIAELDTLnEESYKDSTLIMQLLRDNLTLWTS#.qg#eG#Ae#q##...........

261   268
|------|
AAEGEAPK

........
```
